# Predictive Lung- and Spleen-Targeted mRNA Delivery with Biodegradable Ionizable Lipids in Four-Component LNPs

**DOI:** 10.3390/pharmaceutics17040459

**Published:** 2025-04-02

**Authors:** Juan Heredero, Álvaro Peña, Esther Broset, Beatriz Blandín, Diego de Miguel, Teresa Alejo, Alfonso Toro, Elena Mata, Alexandre López-Gavín, Ana Gallego-Lleyda, Diego Casabona, Verónica Lampaya, Ana Larraga, Esther Pérez-Herrán, David Luna, Irene Orera, Eduardo Romanos, Alba García, Juan Martínez-Oliván, Javier Giménez-Warren

**Affiliations:** 1 Certest Pharma, Certest Biotec S.L., San Mateo de Gállego, 50840 Zaragoza, Spain; jheredero@certest.es (J.H.); apena@certest.es (Á.P.); ebroset@certest.es (E.B.); bblandin@certest.es (B.B.); dmiguel@certest.es (D.d.M.); talejo@certest.es (T.A.); atoro@certest.es (A.T.); emata@certest.es (E.M.); alopez@certest.es (A.L.-G.); agallego@certest.es (A.G.-L.); dcasabona@certest.es (D.C.); vlampaya@certest.es (V.L.); alarraga@certest.es (A.L.); eperez@certest.es (E.P.-H.); dluna@certest.es (D.L.); 2 Department of Theoretical Physics, Faculty of Science, University of Zaragoza, Pedro Cerbuna s/n, 50009 Zaragoza, Spain; 3 Institute for Biocomputation and Physics of Complex Systems (BIFI), University of Zaragoza, Mariano Esquillor s/n, 50018 Zaragoza, Spain; 4 Proteomics Research Core Facility, Aragon Health Sciences Institute (IACS), 50009 Zaragoza, Spain; iorera.iacs@aragon.es; 5 Medical Imaging and Phenotyping Core Facility, Aragon Health Sciences Institute (IACS), 50009 Zaragoza, Spain; eromanos.iacs@aragon.es (E.R.); agarciagil.iacs@aragon.es (A.G.)

**Keywords:** mRNA, ionizable lipids, lipid nanoparticles, extrahepatic delivery, lung targeting, spleen targeting, four-component LNPs, protein corona

## Abstract

**Background/Objectives**: Lipid nanoparticles (LNPs) are leading mRNA delivery vehicles, with ionizable lipids (ILs) as their key component. However, the relationship between the IL structure and LNP endogenous organ-targeting is not well understood. In this study, we developed a novel library of biodegradable ILs featuring beta-propionate linkers, which, when incorporated into a four-component LNP formulation, show excellent extrahepatic selectivity and high protein expression. **Methods**: We explored the impact of structural modifications in the hydrophobic chains and polar-head groups in the ILs while keeping the linkers unchanged. In vivo results were evaluated to examine how structural changes influence the biodistribution to spleen or lungs. LNP formulations were assessed for their protein expression levels and organ-specific targeting. Additionally, protein corona formation by the best-performing LNPs was examined to provide further mechanistic insights. **Results**: Organ targeting was significantly influenced by structural changes in the ILs, allowing for precise control of the biodistribution between the spleen and lungs. Branched hydrophobic chains demonstrated a higher propensity for spleen targeting, while modifications in the polar-head group could drastically shift biodistribution from the lung to the spleen. This led to the identification of LNPs’ zeta potential as a key determinant of their extrahepatic targeting properties. Notably, ionizable lipid A3T2C7, also known as CP-LC-1495, displayed strong lung selectivity (97%) and high protein expression in lung tissue (1.21 × 10^8^ p/s). Similarly, several promising candidates for spleen-targeting LNPs displayed protein expression levels exceeding 1 × 10^7^ p/s (selectivity >80%). **Conclusions**: This study elucidates the structure–function relationships of ILs in passive organ-specific mRNA delivery, highlighting how the fine-tuning of hydrophobic chains, polar-head groups, and surface charge (zeta potential) allows for the precise control of LNP endogenous biodistribution, a mechanism influenced by protein corona formation. These findings enable the rational design of targeted LNP systems, enhancing their therapeutic potential for specific organs, such as the spleen and lungs.

## 1. Introduction

Messenger RNA (mRNA) therapeutics have demonstrated significant potential in treating cancer, genetic disorders, and infectious diseases, as exemplified by the approval of COVID-19 mRNA or respiratory syncytial virus (RSV) vaccines [1,2,3,4]. A critical factor in their success is the use of lipid nanoparticles (LNPs), which are among the most advanced and effective delivery systems for nucleic acid therapeutics. LNPs are formulated with a lipid mixture that usually includes an ionizable lipid, cholesterol, a phospholipid, and a polyethylene glycol (PEG)–lipid [5,6]. However, systemic administration of LNPs leads to predominant mRNA expression in the liver, highlighting the need for novel strategies capable of efficiently targeting extrahepatic tissues for therapeutic applications beyond liver-related diseases or vaccines [7,8,9].

Several approaches have been explored for achieving organ-specific mRNA-LNP delivery beyond the liver, focusing on passive (endogenous) and active (ligand-mediated or exogenous) targeting strategies [10]. Active targeting strategies rely on the addition of targeting ligands to the LNP surface, such as antibodies, peptides, or small molecules, to direct the LNP toward certain cell receptors [11]. Conversely, passive targeting depends on the meticulous design of LNPs, adjusting lipid ratios or incorporating additional lipids to promote the formation of a specific endogenous protein corona upon their introduction into the bloodstream. This protein corona effect has been attributed to different LNP properties such as size, apparent pKa, zeta potential, and PEGylation [12,13]. The passive targeting strategies are particularly promising, as they leverage a controlled structure without the need for additional ligands or modifications, hence reducing costs and regulatory hurdles and simplifying the scale-up process [14].

A well-established approach for passive targeting involves adding a fifth permanently charged lipid—cationic for lung targeting or anionic for spleen targeting—to enable selective organ targeting (SORT) [15,16]. However, this strategy has resulted in limited protein expression levels in targeted organs, indicating potential for further optimization. In a previous study, we refined this approach by developing a combination of an ionizable lipid with its permanently cationic counterpart, which significantly enhanced their efficiency in the lung [17]. Despite this improvement in expression, a key limitation remains, as cationic lipids are associated with relative toxicity in vivo [15,18]. To address this issue, we hypothesized that using rationally designed tissue-specific ionizable lipids rather than permanently cationic or anionic lipids could enhance extrahepatic mRNA delivery while maintaining biocompatibility.

To test our hypothesis, we employed our previously established synthetic method [17], in which we synthesized and evaluated a library of over 1500 novel ionizable lipids in LNPs. This initial evaluation, conducted via intramuscular injection, assessed in vivo performance and identified several promising candidates across different cargo types, including circRNA [19], and demonstrated efficacy with a SARS-CoV-2 vaccine [20]. Building upon this extensive dataset, we conducted a targeted secondary screening process using the zeta potential and LNP in vivo expression efficiency as criteria to identify candidates suitable for lung-targeted delivery. This approach allowed us to focus on a specific lipid family that shows selective extrahepatic delivery.

This lipid family features beta-propionate linkers that are stable at neutral pH but that become labile under acidic conditions (pH 5.3) [21], potentially enhancing LNP endosomal escape. We evolved this lipid family and obtained selective targeting to pulmonary or splenic tissues through structural modifications in four-component standard formulations without the need for permanently charged lipids. While other studies have recently explored organ-specific passive targeting to the spleen and lungs with four-component LNPs [22,23,24], to our knowledge, this is the first to use biodegradable beta-propionate-containing ionizable lipids within a single lipid library and the standard four-lipid composition ratio. These results prompted us to explore whether refining the lipid library could enable the prediction of lung and spleen targeting based on the ionizable lipid structure and LNP physicochemical properties.

We conducted in vivo studies on a diverse set of ionizable lipids, systematically varying their polar-head groups and hydrophobic chains. This approach allowed us to establish key design principles linking the zeta potential to selective organ targeting. As a result, we identified promising LNP candidates with enhanced specificity for lung and spleen delivery. Subsequently, the protein corona behavior of the organ-specific LNPs was evaluated through proteomic analysis. Notably, certain proteins were found to be characteristically abundant in the coronas of lung-specific LNPs, showing a correlation between protein adsorption patterns and tissue tropism. Additionally, we achieved superior total flux signals and selectivity compared with previously reported charged lipid-targeting strategies [16] for lung- (97.8% selectivity) and spleen (95.6% selectivity)-selective delivery, highlighting the enhanced targeting effectiveness of this ionizable lipid library. These findings establish a framework for predicting and optimizing lung and spleen targeting through rational ionizable lipid design and LNP physicochemical properties.

## 2. Materials and Methods

### 2.1. Chemicals

The chemical reagents used in this study were sourced from a variety of suppliers, including Sigma Aldrich (Burlington, MA, USA), Tokyo Chemical Industry (TCI, Tokyo, Japan), Fluorochem Ltd. (Hadfield, UK), and Ambeed (Arlington Heights, IL, USA). Cholesterol and 18:1 PA were both purchased from Sigma Aldrich (Burlington, MA, USA). The 1,2-dioleoyl-sn-glycero-3-phosphoethanolamine (DOPE) was obtained from Avanti Polar Lipids (Alabaster, AL, USA), while the 1,2-dimyristoyl-rac-glycero-3-methoxypolyethylene glycol-2000 (DMG-PEG 2000) was acquired from Cayman Chemicals (Ann Arbor, MI, USA). DLin-MC3-DMA (MC3) was purchased from Broadpharm (San Diego, CA, USA). The ionizable lipid DOTAP and 1-Octylnonyl 8-((2-hydroxyethyl) (6-oxo-6-(undecyloxy)hexyl)amino)octanoate (SM-102) were sourced from BOC Sciences (Shirley, NY, USA).

### 2.2. mRNA Synthesis

The synthesis of Firefly luciferase mRNA was carried out through in vitro transcription using T7 RNA polymerase and a linearized DNA template. This template comprised the luciferase coding sequence, flanked by 5′- and 3′-UTRs, and included a 100A polyA tail at the 3′-end [25]. The transcription reaction mixture contained ATP, GTP, CTP, N1-methylpseudouridine, and co-transcriptional CAP AG. Following transcription, the mRNA was purified using affinity chromatography with a POROS Oligo(dT)25 column (ThermoFisher Scientific, Waltham, MA, USA). The concentration of the purified mRNA was measured by absorbance at 260 nm, adjusted to 1 mg/mL, aliquoted, and stored at −80 °C. To ensure the quality of the sample, automated electrophoresis was performed using an Agilent 2100 Bioanalyzer G2938B (Santa Clara, CA, USA) to confirm the integrity of the mRNA prior to storage.

### 2.3. General Method for the Synthesis of Ionizable Lipids and Characterization

Ionizable lipids were prepared through a tandem one-pot reaction. The process began by dissolving thiolactone-sulfur derivatives (0.15 mmol; 1 equiv.) and acrylate (0.15 mmol; 1 equiv.) in 300 µL of tetrahydrofuran (THF) at room temperature, followed by the addition of amine (0.15 mmol; 1 equiv.). After two hours, THF was evaporated under vacuum, and the crude products obtained were directly used for ex vivo screening.

The synthesized lipids were subjected to analysis using high-performance liquid chromatography (HPLC) with a charged aerosol detector (CAD) and further characterized by mass spectrometry. Proton nuclear magnetic resonance (1H NMR) spectra were acquired using a Bruker 400 MHz NMR spectrometer (Billerica, MA, USA). Additionally, HPLC-CAD-MS analysis was conducted with a Vanquish HPLC system equipped with a Vanquish Charged Aerosol Detector and an ISQ EC Single Quadrupole Mass Spectrometer (ThermoFisher Scientific).

### 2.4. Characterization of Ionizable Lipid A3T2C7 (CP-LC-1495)

CP-LC-1495 was purified using a CombiFlash NextGen 300+ system with gradient elution, transitioning from 100% dichloromethane to 30% of an 80/20/1 DCM/MeOH/NH4OH (aq.) solution. Structural confirmation was performed via mass spectrometry (MS) and nuclear magnetic resonance spectroscopy (NMR). MS-QDa: theoretical [M + H]^+^ = 960.62, experimental [M + H]^+^ = 960.56; ^1^H NMR (400 MHz, CDCl3) δ: 6.64 (t, J = 4.9 Hz, 1H), 6.41 (m, 1H), 4.56 (q, J = 7.3 Hz, 1H), 4.08 (m, 4H), 4.00 (m, 2H), 3.30–3.15 (m, 5H), 2.76 (m, 7H), 2.67 (t, J = 6.8 Hz, 2H), 2.57 (m, 10H), 2.23 (t, J = 7.6 Hz, 2H), 2.08 (m, 4H), 1.93 (m, 1H), 1.77 (m, 2H), 1.59 (dt, 8H), 1.44 (m, 2H), 1.30 (m, 28H), 0.88 (m, 12H).

### 2.5. Lipid Nanoparticle Formulation

LNPs were prepared via microfluidics using an ethanol-based lipid solution containing ionizable lipid, DOPE (helper lipid), cholesterol, and DMG-PEG2000 (50:10:38.5:1.5 molar ratio) [26]. This was combined with an aqueous phase of mRNA in 10 mM citrate buffer (pH 4) at a molar N/P ratio of 6. Mixing was performed using a NanoAssemblr^®^ Ignite^TM^ device (Precision NanoSystems, Vancouver, BC, Canada) with a 3:1 flow-rate ratio (FRR) and 12 mL/min total flow rate (TFR). Post-formulation, LNPs were dialyzed overnight against pH 8 Tris buffer with 15% sucrose. The final product was adjusted to 100 µg/mL mRNA, filtered (0.22 µm), and stored at 4 °C.

### 2.6. Formulation of SORT LNPs

Five-component LNPs (cationic DOTAP or anionic 18:1 PA variants) were synthesized via microfluidics. Ethanol-based lipid phases included charged lipid, ionizable lipid, DOPE, cholesterol, and DMG-PEG2000 at molar ratios of 50:11.9:11.9:23.8:2.4 for DOTAP LNPs and 10:21.4:21.4:42.9:4.3 for 18:1 PA LNPs [16]. These were mixed with mRNA in citrate buffer (pH 4) at an N/P ratio of 10 (*w*/*w*) using the NanoAssemblr^®^ Ignite^TM^ (FRR 3:1, TFR 12 mL/min). Dialysis, filtration, and storage conditions were the same used in the Section 2.5.

### 2.7. Characterization of LNPs

Hydrodynamic size, zeta potential, and polydispersity index (PDI) were measured using a Malvern Zetasizer^®^ Advance Lab Blue Label (Malvern, UK). mRNA encapsulation efficiency was determined via Quant-IT^®^ Ribogreen assay. Full characterization data are provided in the Supplementary Materials.

### 2.8. Animals

All animal experiments were performed in compliance with European and national regulations for the protection of experimental animals (Directive 2010/63/EU and Spanish Royal Decree 53/2013). The experimental protocols were reviewed and approved by the Ethics Committee for Animal Experiments at the University of Zaragoza (PI07/23). The study adhered to the principles of the 3Rs (Replacement, Reduction, and Refinement) to minimize both animal suffering and the number of animals used.

Female BALB/cAnNRj mice, aged 8 to 10 weeks, were purchased from Janvier Labs. Upon arrival at the Centro de Investigaciones Biomédicas de Aragón (Zaragoza, Spain; reference ES 50 297 0012 01), the mice were housed under specific-pathogen-free (SPF) conditions and allowed a one-week acclimation period. The housing environment was carefully controlled, maintaining a temperature of 20–24 °C, humidity levels between 50–70%, light intensity at 60 lux, and a 12-h light–dark cycle. Mice had access to food and water ad libitum.

Sample sizes for each study were determined through statistical power calculations to ensure reliable and reproducible results while minimizing the number of animals used. Before starting the experiments, all animals were monitored for signs of illness, injury, or abnormal behavior that could disqualify them from participation; no animals met these exclusion criteria. To reduce bias, animals were randomly assigned to experimental groups.

### 2.9. Ex Vivo Imaging

Animals were injected via the tail vein with 0.5 mg/kg of luciferase-encoding mRNA-LNPs, which were diluted in Tris buffer containing 15% sucrose to a final volume of 250 µL and administered using a 27G needle.

Four hours after the injection, mice were anesthetized in an inhalation chamber with 4% isoflurane (IsoVet), and anesthesia was maintained at 1.5% isoflurane throughout the procedure. D-luciferin (12507, Quimigen, Alverca do Ribatejo, Portugal), diluted in PBS, was then administered intraperitoneally at a dose of 150 mg/kg. Twenty minutes after the luciferin injection, the mice were euthanized, and their liver, spleen, lungs, heart, kidneys, and intestine were aseptically harvested. Ex vivo luminescence images of the collected organs were acquired using the IVIS Lumina XRMS Imaging System (Revvity Inc., Waltham, MA, USA) following the manufacturer’s instructions.

### 2.10. Isolation of Plasma Proteins Adsorbed to LNPs

LNPs were first diluted to a final lipid concentration of 1 g/L in 1× PBS before being mixed with human plasma at a 1:1 volume ratio. The mixture was incubated for 1 h at 37 °C. Plasma samples were provided by the Biobank of Aragon under standard operating procedures approved by the Ethical and Scientific Review Boards within the framework of the Aragon Health Sciences Institute.

After incubation, the LNP/plasma mixture was layered onto a 0.7 M sucrose cushion in equal volume and centrifuged at 15,000× *g* at 4 °C for 1 h. This process was repeated twice more for a total of three washes. The resulting pellet was resuspended in TRIS-HCl (150 mM pH 8), and excess lipids were removed through TCA/acetone precipitation.

The cleaned pellet was then resuspended in 2× Laemmli buffer and loaded onto a 12% Mini-PROTEAN TGX Precast Protein Gel. The gel was run at 90 V until the proteins migrated approximately 1 cm into the gel. To fix and visualize protein bands, the gel was stained with SimplyBlue Safe Stain for 1 h. Protein bands were excised using a sterile razor blade, placed in ddH2O, and stored at 4 °C until further analysis by mass spectrometry.

### 2.11. Mass Spectrometry Identification of Protein Coronas

The digestion of the polyacrylamide bands was performed in an automatic digester (Intavis, Bioanalytical Instruments, Cologne, Germany). Bands were washed with water, ammonium bicarbonate (100 mM), and acetonitrile (ACN). The samples were reduced by incubation with DTT (10 mM) at 60 °C for 45 min and alkylated by incubation with iodoacetamide (50 mM) at room temperature for 30 min. Digestion was carried out with trypsin overnight at 37 °C (300 ng, Trypsin Gold, Promega, WI, USA). Digestion was stopped by adding 0.5% trifluoroacetic acid and the peptides were extracted sequentially with increasing concentrations of ACN in water.

Proteins were identified in a hybrid trapped ion-mobility quadrupole time-of-flight mass spectrometer (TIMS TOF Flex, Bruker Daltonics, Bremen, Germany) coupled online to an EvoSep ONE liquid chromatograph (EvoSep, Odense C, Denmark). Peptides (200 ng) were loaded onto the EvoSep ONE chromatograph using the 30 SPD (samples per day) chromatographic method.

Peptides were separated on a C18 column (15 cm × 150 μm, 1.5 μm, Evosep) using a linear 44-min gradient and a cycle time of 48 min at a constant flow rate of 0.5 uL/min. Column temperature was set at 40 °C. Data were acquired using data-dependent acquisition mode with PASEF (parallel accumulation serial fragmentation). MS data were collected over an *m/z* range of 100 to 1700 and a mobility range of 0.60–1.60 V s/cm^2^. During each MS/MS data collection, each cycle was 1.1 s and included 1 MS and 10 PASEF MS/MS scans.

Protein identifications were carried out with Bruker ProteoScape^TM^ (BPS) software (version 2025b) and were searched against the Uniprot human protein database plus sequences of known contaminants concatenated to a decoy database in which the sequence for each entry in the original database was reversed using ProLuCID algorithm (version 2015). Twenty ppm precursor tolerance and 30 ppm fragment ion tolerance were used. Two missed cleavages were accepted. Carbamidomethylation (+57.02146) of cysteine was considered a static modification. TIMScore was enabled to allow the use of the peptide Collisional Cross Section (CCS) during the scoring process. These search results were validated, assembled and filtered using the DTASelect program (version 1.2.0) with a false discovery rate of 1% at the protein level in all analysis.

### 2.12. Bioinformatic Analysis of Protein Coronas

Mass-spectrometry-identified proteins were initially filtered to exclude non-human contaminating proteins. The physicochemical properties of the remaining proteins in each sample were computed using the ProtParam module of Biopython [27]. For protein abundance quantification, the data were first filtered to remove proteins with low coverage, and abundance percentages were then calculated using the Normalized Spectral Abundance Factor (NSAF) method.

To identify proteins relevant for lung or spleen targeting, we applied quantitative selection criteria based on their relative abundance across different organ experiments. First, protein concentrations were normalized so that the total abundance across all experiments summed to 100%. Then, selection criteria were established as follows: For lung-enriched proteins: (i) The protein must have a normalized NSAF abundance greater than 0.0015 in at least one lung-targeting LNP sample. (ii) The cumulative normalized abundance across the three lung experiments must exceed 75. (iii) The protein must exhibit a minimum normalized abundance of 20 in each of the three lung-targeting LNPs. (iv) The protein must not exceed a normalized abundance of 20 in any non-lung targeting LNP. For spleen-enriched proteins, the criteria were the following: (i) The protein must have a minimum normalized NSAF value of 0.00075 in at least one spleen-targeting LNP. (ii) The total normalized abundance across the spleen-targeting LNP samples must be at least 50%. (iii) The protein must exhibit a minimum normalized abundance of 20 in each spleen-targeting LNP. (iv) The protein must not exceed a normalized abundance of 30 in any non-spleen targeting LNP.

## 3. Results and Discussion

### 3.1. Strategy for the Identification of the Lung-Targeting Lipid Library

**Figure 1:**
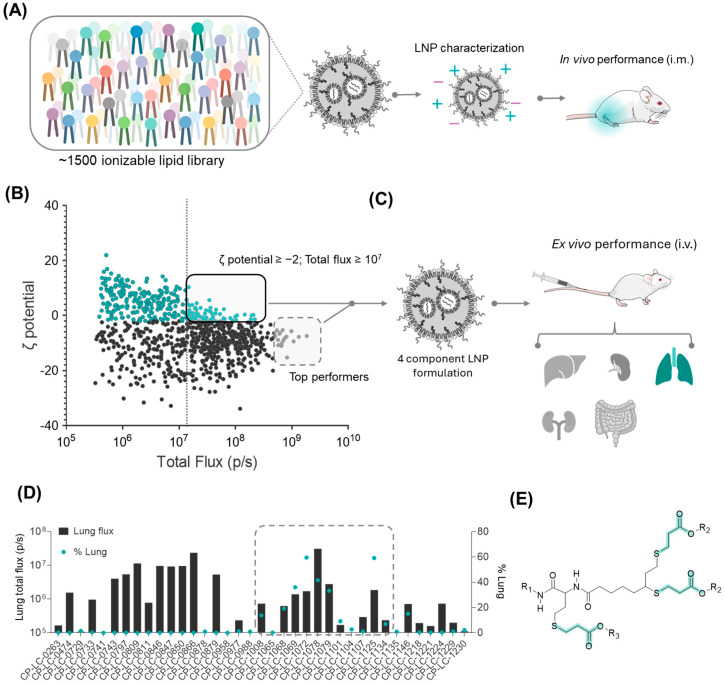
Retrospective lipid library analysis for the screening and selection of ionizable lipids with lung-targeting LNPs. (**A**) Scheme of ionizable lipid screening formulated in LNPs administered via in vivo intramuscular (i.m.) injection, where mice received mRNA-Luc-loaded LNPs at a dose of 0.05 mg/kg mRNA. (**B**) Graphic of the ζ potential and total flux of ~1500 ionizable lipids from the intramuscular screening. LNPs with equal or greater than zero potential are illustrated with green color. Ionizable lipids contained in LNPs within boxes were selected as potential candidates for lung targeting. (**C**) Illustration of the lung-targeting screening in mice. (**D**) Graphic of the ex vivo total bioluminescence flux quantification and selectivity in the lung for selected ionizable lipids. Mice were intravenously (i.v) injected with mRNA-Luc-loaded LNPs at an mRNA dose of 0.5 mg/kg (*n* = 2 biologically independent samples). Data are presented as mean values. (**E**) General lipid structure identified for extrahepatic targeting containing up to three beta-propionate linkers; beta-propionate linkers are highlighted in blue.

One of the key challenges in mRNA therapeutics is targeting LNPs beyond the liver, as conventional formulations are prone to being expressed in the liver because of their interaction with the protein apolipoprotein E (ApoE) [28]. In our previous study, we conducted a large-scale screening of 1500 ionizable lipids using our STAAR platform (Figure 1A), where each lipid was formulated into a standard LNP composition (50:38.5:10:1.5 of ionizable lipid:cholesterol:DOPE:DMG-PEG; molar N/P ratio of 6) [17,26] encapsulating luciferase mRNA and administered intramuscularly in mice (0.05 mg/kg of mRNA). This screening provided us with a robust dataset of ionizable lipid performance in the muscle, which we anticipated would likely lead to strong expression in the liver.

However, given the known influence of LNP surface charge on protein corona formation [12,29], we hypothesized that some of our ionizable lipids forming LNPs with zeta potential values outside the typical range for liver targeting might express preferentially in other organs. To test this hypothesis, we retrospectively re-evaluated these lipids, selecting the screened candidates that yielded LNPs with physicochemical properties deviating from the liver-targeting norms. We specifically focused on the zeta potential as this reflects the overall surface charge, which can influence interactions with serum proteins, the biodistribution, and passive organ targeting.

Thus, for this selection, we applied two criteria. First, we focused on LNPs with a zeta potential equal to or greater than −2 mV, inspired by previous findings that suggested that an increased surface charge could enhance lung targeting [10,12]. Second, we included LNPs that demonstrated strong protein expression, specifically those exceeding a total flux of 1 × 10^7^ p/s in the original intramuscular screening, ensuring that only high-performing candidates were considered (chemical structures can be found in Figure S1). Additionally, we incorporated thirteen lipids that exhibited exceptionally high protein expression despite having more negative zeta potentials, allowing us to explore potential outliers with unique targeting properties (Figure 1B).

After intravenous administration of LNPs formulated with the selected ionizable lipids and encapsulating luciferase mRNA, ex vivo analyses revealed that, while most of the selected ionizable lipids still showed liver tropism, eight of them demonstrated lung-selective expression exceeding 10% selectivity (Figure 1C,D). Interestingly, six of these eight lipids shared a distinct structural feature: the presence of three beta-propionate linkers and *N*,*N*-dimethylethylenediamine (A1) as the polar head (Figure 1E). Recognizing the potential of this family of lipids as new candidates for pulmonary mRNA therapeutics, we decided to conduct further investigations into their physicochemical and structural properties to better understand their lung-selective behavior.

### 3.2. Impact of Hydrophobic Tail Modifications in the Ionizable Lipids on Extrahepatic Delivery Efficiency

Having identified a common backbone in the structure of our lipids, we then performed structure–activity studies to explore how chemical modifications in specific moieties of these lipids could affect their extrahepatic delivery performance, particularly in their targeting to the lungs.

**Figure 2:**
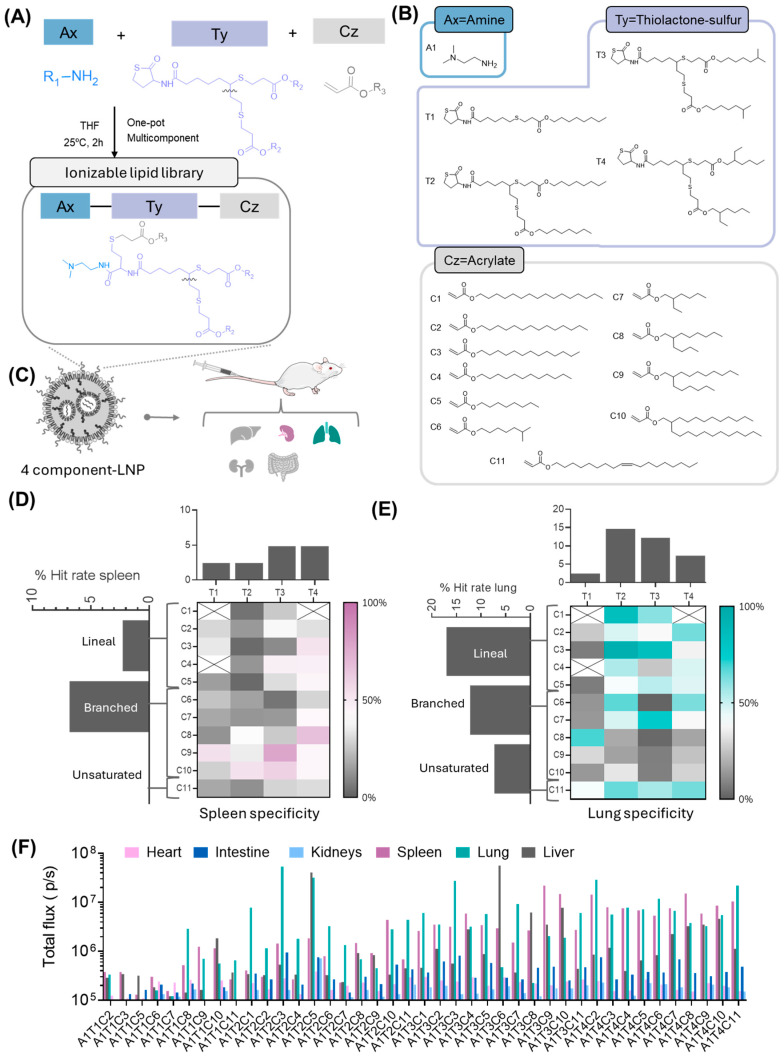
Combinatorial synthesis and organ-specific targeting of beta-propionate ionizable lipids. (**A**) Scheme describing the steps for the synthesis of the ionizable lipids. (**B**) Structures of the 4 thiolactone-sulfur (Ty) derivatives, 11 acrylates (Cz) and amines (Ax) used in the combinatorial synthesis. (**C**) Illustration of the lung and spleen targeting. Mice were i.v. injected with mRNA-Luc-loaded LNPs at an mRNA dose of 0.5 mg/kg. (**D**) Heat map depicting the spleen specificity of ionizable lipids, correlated with the hit rate of Cz hydrophobic tails and Ty in spleen tissue. (**E**) Heat map depicting the spleen specificity of ionizable lipids, correlated with the hit rate of Cz hydrophobic tails and Ty. (**F**) Quantified luciferase expression in spleen, liver, lung, heart, intestine and kidneys. (**D**,**E**) An “X” indicates that the LNPs precipitated and were not used in the study. Mice were i.v. injected with mRNA-Luc-loaded LNPs at an mRNA dose of 0.5 mg/kg (*n* = 2 biologically independent samples). Data are presented as mean values.

Hydrophobic tail regions are known to play a pivotal role in determining the LNP biodistribution, impacting factors such as lipid packing, LNP stability, and their cellular uptake in target tissues [6,30,31]. Thus, in this study, we modified the tail structure of selected lipids by introducing varying lengths, degrees of saturation, and branching patterns, assessing how these changes affected extrahepatic targeting, specifically their lung and spleen selectivity (Figure 2A,B).

Following our previously reported screening approach [17], which relied on a one-pot reaction of a thiolactone-sulfur derivative (Ty) with an amine (Ax) and an acrylate (Cz) (Figure 2A), we synthesized a selection of 44 lipids (Table S1) with purities of over 80%. We included a comparison between the crude and purified products in HPLC-CAD (Figure S4) by combining four different thiolactone-sulfur derivatives (T1–T4, bearing linear and branched aliphatic chains) with eleven different acrylates (C1–C11, which contained linear, unsaturated, or branched tails). Along with two biodegradable amines (derived from Ax and Ty), the resulting final lipid structure contained at least two beta-propionate linkages (derived from Ty and Cz) which are known to have superior biodegradability [21] in mildly acidic environments, potentially improving endosomal escape [32]. For this initial assessment, the amine selected was the same as the one identified in the previous section, namely *N*,*N*-dimethylethylenediamine (A1, Figure 2B). The crude synthesized lipids were then formulated into LNPs and injected intravenously into mice. The resulting LNPs showed encapsulating efficiencies exceeding 90%, hydrodynamic diameters ranging from 70 to 110 nm, and a narrow size distribution (PDI < 0.3). No clear trends were observed in the LNP encapsulation efficiencies, sizes, or PDIs. However, significant variations in the LNP zeta potential were evident across different ionizable lipid structures, displaying positive, negative, and near-neutral values (Table S2).

To evaluate the organ selectivity ex vivo, we defined organ-specific hit rates based on a 50% protein expression threshold. Consequently, an LNP was deemed organ-selective if at least 50% of its total expression occurred in a single organ (Figure 2D,E). Notably, 36.6% of the lipids demonstrated selectivity for lung tissue, highlighting that a significant proportion of the tested compounds effectively targeted pulmonary cells. Remarkably, our screening revealed that as few as 7.3% of the ionizable lipids tested demonstrated selectivity for hepatic tissue. This key finding establishes a foundation for developing novel strategies for extrahepatic delivery, particularly considering the liver’s predominant role in mRNA-LNP biodistribution (Table S3).

Closer examination of the chemical structures of the tested tails revealed that T1 derivatives barely achieved lung selectivity (only one ionizable lipid in that group; 2.4% hit rate), suggesting that a minimum level of branching is required to enhance targeting to lung tissue (Figure 2E). This lack of selectivity in T1 may also stem from the removal of one of the biodegradable beta-propionate linkages in its final structure, which could impact both its stability and biodistribution. In contrast, the branched thiolactone-sulfur derivatives (T2, T3, T4) were designed to contain eight carbons post-ester linkage, making them structural isomers and allowing us to assess how tail structures influence extrahepatic delivery. Among these, T2 (linear n-octyl chains) exhibited the highest proportion of lung-selective ionizable lipids (14.6% hit rate), followed by T3 (12.2% hit rate), with T4 showing the least lung selectivity of the three groups (7.3% hit rate) (Figure 2E). This suggests a structure–activity relationship in which the linear n-octyl chains of T2 offers an advantageous configuration for lung targeting, while the further branching in T3 and T4 appears less effective for this purpose.

Interestingly, during the ex vivo experiments, in addition to analyzing lung tissue, we also evaluated other organs, including the liver, kidney, intestine, and spleen. Notably, 14.6% of the evaluated lipids demonstrated selectivity for the spleen (Figure 2D). Further analysis of the chemical structures revealed that increased branching within the T3 and T4 regions correlated with a shift in selectivity toward splenic tissue. This data suggests that, while the less-branched chains in the thiolactone-sulfur derivatives favor lung targeting, increased branching shifts the selectivity toward spleen. This shift in organ targeting observed with increasing lipid branching likely stems from changes in the lipid nanoparticle (LNP) physicochemical properties and their subsequent influence on protein corona formation. Less-branched chains, which have a more linear and compact structure, may promote a corona enriched with lung-specific proteins, enhancing pulmonary targeting. In contrast, branching introduces steric bulk and increases the hydrophobicity of the lipid tails, leading to ionizable lipids with a more conical shape and LNPs with reduced surface-charge density and a different packing efficiency [30]. These branched lipids likely recruit a spleen-favoring protein corona, driving uptake by spleen cells, a trend supported by findings on corona-mediated biodistribution [13]. This interplay between branching, LNP structure, and corona composition offers a potential pathway for designing organ-targeted delivery systems.

A similar pattern of branching impacting organ selectivity was observed in the aliphatic chains of the acrylate component (Cz). Although this moiety appears to have a smaller influence on selectivity compared with the thiolactone-sulfur derivatives, five out of six lipids exhibiting high spleen selectivity (>50% total protein expression) contained branched acrylates (Figure 2D). This finding reinforces the notion that increased branching within lipid structures tends to enhance spleen targeting, highlighting the cumulative effect of branched components in directing organ-specific delivery.

Apart from high percentages for organ selectivity, most of the tested lipids achieved protein expression levels exceeding 1 × 10^6^ p/s in the lungs, underscoring their potential for targeted pulmonary delivery (Figure 2F). Notably, lipid A1T2C3 (also known as CP-LC-1067) stood out with a high protein expression level, reaching 5.32 × 10^7^ p/s. This dual success in selectivity and expression positions CP-LC-1067 as a promising candidate for lung mRNA delivery.

### 3.3. Influence of the Polar Head on Extrahepatic Delivery Efficiency

**Figure 3:**
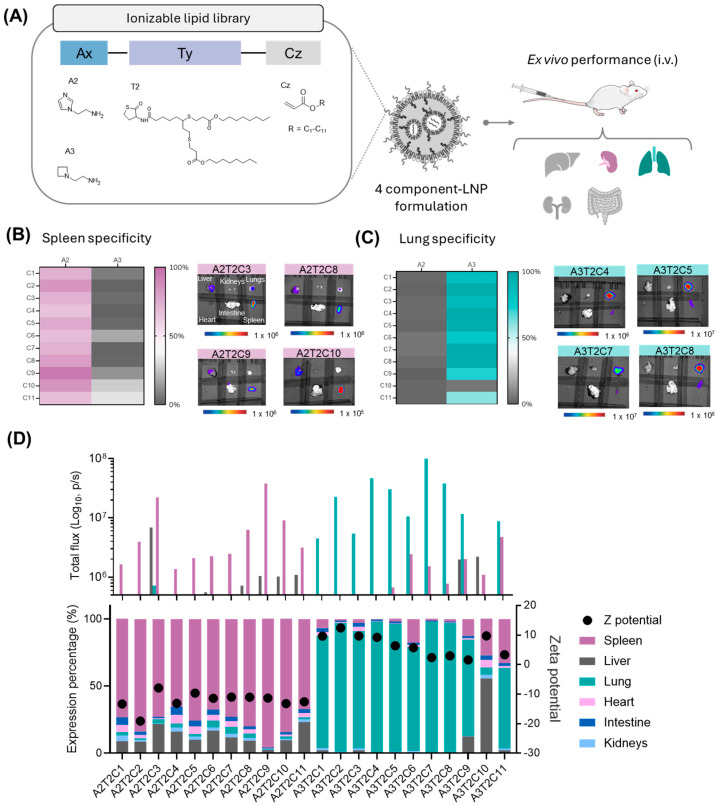
Analysis of the ionizable lipid polar-head influence on organ-specific targeting. (**A**) Structures of the thiolactone-sulfur (Ty) derivatives, acrylates (Cz) and amines (Ax) employed for the ionizable lipid synthesis. These ionizable lipids were incorporated into LNPs for the evaluation of organ targeting. (**B**) (Left) Heat map depicting the spleen specificity of the ionizable lipids. (Right) Representative ex vivo bioluminescence images of mouse organs. (**C**) (Left) Heat map depicting the lung specificity of the ionizable lipids. (Right) Representative ex vivo bioluminescence images of mouse organs. The organs are arranged in two rows: the top row (from left to right) includes the liver, kidneys, and lungs, while the bottom row (from left to right) includes the heart, intestines, and spleen. (**D**) (Top) Total flux (p/s) in the six main organs obtained for the LNP formulation of each lipid candidate upon i.v. administration and subsequent ex vivo analysis. (Bottom) Stacked bar chart depicting the percentage expression in each organ for the lipid candidates upon ex vivo analysis. Zeta potential values are shown as black dots within the graph. Mice were i.v. injected with mRNA-Luc-loaded LNPs at an mRNA dose of 0.5 mg/kg (*n* = 2 biologically independent samples). Data are presented as mean values.

Recognizing the potential of this family of lipids, we sought to further explore structural variations within the ionizable lipid family by modifying its polar-head group while keeping the T2 component constant, aiming for a more refined lung- or spleen-targeting efficiency. For this study, we selected two polar heads. The first one incorporated an imidazole substituent as the ionizable moiety, which was chosen based on previous reports indicating its potential for spleen targeting [33,34]. The second polar head included an azetidine residue, which features an ionizable amine within a cyclic structure. This latter polar head was selected as it represents the closest cyclic analog to amine A1 (Figure 3A).

Applying the same previously established one-pot synthetic method (Figure 2A), 22 new ionizable lipids were synthesized, formulated into LNPs, and tested intravenously in mice by ex vivo luminescence analysis (Figure 3A). Employing the same hit-ratio rule as before (over 50% selective expression in a single organ), a remarkable 95% of the LNPs were found to have extrahepatic selectivity, with only one (A3T2C10) out of the 22 LNPs predominantly expressed in the liver (Table S4; Figure 3B,C). Strikingly, amongst the 95% of extrahepatic-targeted LNPs, there was a strong distinction between both polar heads, as most of the imidazole-based lipids (A2 polar head) showed targeting to the spleen (Figure 3B), a result that aligns with previously reported findings [33], whereas the azetidine lipids (A3 polar head) showed a clear affinity for the lungs (Figure 3C). Zeta potential analysis of the LNPs revealed a distinct pattern that correlated with each polar-head group (Figure 3D). Notably, a clear trend was observed based on the zeta potential, with LNPs exhibiting selectivity for splenic tissue demonstrating a negative zeta potential (range −19 to −9.7 mV; Figure 3D and Table S2), whereas those preferentially targeting pulmonary tissue displayed a slightly positive surface charge (range 0.95 to 3.5 mV; Figure 3D and Table S2). This observed correlation between the zeta potential and organ selectivity further supports the role of surface charge in directing LNP biodistribution, reinforcing the potential of rational lipid design for extrahepatic delivery. This distinction in surface-charge characteristics likely influences the interactions between LNPs and biological components, ultimately affecting their biodistribution and organ targeting.

Beyond selectivity, expression levels are equally critical, as low expression may compromise the therapeutic efficacy of LNPs, making high selectivity alone insufficient for effective organ-targeted delivery. Notably, ionizable lipids with A2 and A3 not only exhibited strong organ specificity but also achieved high luciferase expression levels, with the selective organ total flux surpassing 1 × 10^7^ p/s for nine of the ionizable lipids (Figure 3D). Particularly, lipid A3T2C7 (also known as CP-LC-1495) not only achieved a high level of lung selectivity with a remarkable 97.1% but also showed a strikingly high luminescence signal of 1.21 × 10^8^ p/s, highlighting its potential for targeted lung delivery therapeutics. Moreover, several ionizable lipids exhibited spleen-tissue targeting with high selectivity (>80%) and high efficiency (approximately 1 × 10^7^ p/s; Figure 3D). Additionally, cytotoxicity experiments in relevant lung (A549), spleen (Jurkat), and liver (HepG2) cell lines resulted in cell viabilities above 90% for A549 along with HepG2 and above 75% for Jurkat cells (Figure S3), even when using LNP-mRNA concentrations four times higher than those reported in previous studies [23].

The striking selectivity and efficiency of spleen- and lung-targeting ionizable lipids, coupled with their excellent cytotoxicity profiles, prompted us to further investigate the protein corona’s role in driving organ-specific delivery to the lungs and spleen of the top-performing ionizable lipids.

### 3.4. Proteomic Analysis of Top-Performing LNPs in Extrahepatic Targeting

**Figure 4:**
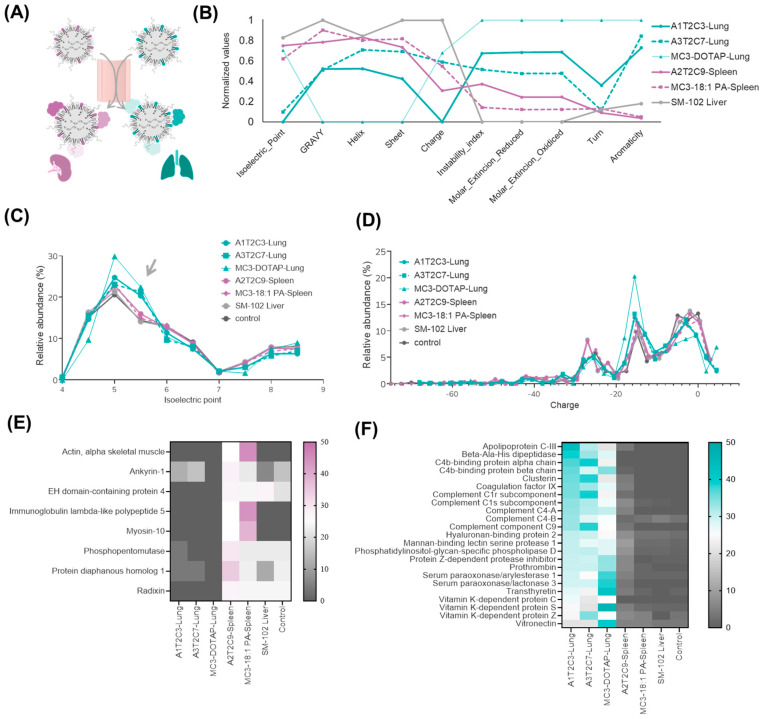
Protein-corona composition of organ-targeting LNPs. (**A**) Schematic illustration of how protein coronas form upon entry into the bloodstream, influencing nanoparticle targeting and directing them to specific organs. (**B**) Normalized physicochemical properties of adsorbed proteins, including their isoelectric point, hydrophobicity (GRAVY), secondary structure elements (helix, sheet, turn), instability index, charge, and aromaticity. (**C**) Distribution of the isoelectric points (arrow) and (**D**) net charge of proteins adsorbed onto lung- and spleen-targeting LNPs. (**E**) Heatmap of enriched proteins in spleen-targeting and (**F**) lung-targeting LNP coronas.

The composition of the protein corona that forms around LNPs upon exposure to biological fluids is a key factor in their organ selectivity and biodistribution [28,35]. Given this well-established relationship, we sought to investigate whether the differences in our extrahepatic lipid library were reflected in distinct protein corona compositions, which could potentially explain their selective accumulation in non-hepatic tissues. With that purpose, we selected and purified the two top-performing ionizable lipids for lung targeting (A1T2C3, also named CP-LC-1067, and A3T2C7, also named CP-LC-1495) and the highest-performing lipid for spleen targeting (A2T2C9, also named CP-LC-1465). As controls, we included MC3-DOTAP and MC3-18:1 PA, well-established SORT formulations known for targeting the lung and spleen, respectively, with previously characterized protein coronas [12]. Additionally, we incorporated liver-targeting LNPs using SM-102 as the ionizable lipid and a control without LNPs. Subsequently, to mimic the in vivo environment, we incubated LNPs with human plasma, allowing the natural formation of a protein corona similar to that occurring in the bloodstream (Figure 4A). After purification and LNP protein-corona isolation, we performed mass-spectrometry-based proteomic analysis to identify and quantify the adsorbed proteins.

Examination of key physicochemical properties of the enriched proteins in different selected LNPs samples, including of the isoelectric point, hydropathy (GRAVY index), secondary structure, charge, and stability, showed that lung-targeting LNPs exhibited a notable increase in charge and hydropathy, while spleen- and liver-targeting LNPs displayed distinct trends in protein stability and secondary structure (Figure 4B). Since zeta potential was identified as a key factor in organ targeting by our ionizable lipid library, we further analyzed its influence on related protein properties such as isoelectric point (pI) and overall charge. We found that in lung-targeting LNPs, the isoelectric point distribution of adsorbed proteins was slightly shifted toward pI ~5.5 compared with liver- and spleen-targeting LNPs (Figure 4C). This may be attributed to their higher zeta potential, which likely influences protein binding. Notably, vitronectin [12], a key mediator of lung uptake, has a pI of ~5.5, suggesting a potential explanation for this enrichment. In contrast, the protein-charge distribution was less affected by lipid composition (Figure 4D). This may be due to the LNP charge being better described as charge density rather than absolute charge. While surface charge impacts protein adsorption, it operates within a complex electrostatic environment, which could explain the subtler differences in protein-charge profiles compared with pI trends. These findings suggest that lipid composition influences not only protein adsorption but also the physicochemical characteristics of the corona, impacting organ-specific delivery.

To further investigate protein-corona-driven targeting, we analyzed the top 30 most abundant proteins in lung- and spleen-targeting LNP coronas (Tables S5–S10). Additionally, to provide a clearer interpretation of the results, we focused on proteins that were highly enriched in one organ-specific LNP corona while being absent or barely detected in the other (Figure 4E,F). Results showed that the predominant proteins absorbed in spleen-targeting LNPs are actin, myosin, and the immunoglobulin lambda-like protein. These proteins exhibit a low correlation and are typically nonspecific and are often found in negative mass spectrometry controls. Therefore, the mechanism underlying spleen targeting remains unclear. However, the protein-corona composition of lung-targeting LNPs was clearly distinct from that of liver- and spleen-targeting LNPs. Notably, vitronectin and prothrombin, both extensively reported as key protein-corona components influencing lung targeting, were among the most enriched proteins [12,13,27]. These findings suggest that lung-targeting LNPs in our ionizable lipid library follow previously described mechanisms of proteincorona-mediated lung selectivity.

## 4. Conclusions

The safe and efficient extrahepatic delivery of mRNA therapies remains a significant challenge. While fine-tuned LNPs have demonstrated high potential, a critical gap persists in understanding the structure–activity relationships of ionizable lipids when designing LNPs [35]. In this study, we demonstrate how structural modifications of ionizable lipids can precisely control organ-specific targeting. Notably, our approach did not require permanently charged lipids; instead, we achieved selective pulmonary and splenic targeting using only standard four-component LNP formulations. Thus, we employed a one-pot synthesis strategy based on our previously established method [35], enabling the evaluation of over 1500 novel ionizable lipids in LNP formulations. The zeta potential of lipid nanoparticles (LNPs) was hypothesized to be a critical factor in their ability to target extrahepatic tissues. From this, we selected a specific lipid structure featuring up to three beta-propionate linkers as biodegradable moieties, which guided our further lipid screening.

Building on this, we synthesized a novel library of beta-propionate-containing ionizable lipids and conducted in vivo testing. Our results revealed that branched hydrophobic chains enhance spleen targeting, while modifications in the polar-head group significantly alter biodistribution, favoring either the lungs or spleen. Specifically, spleen-targeting LNPs exhibited a negative zeta potential, whereas lung-targeting LNPs displayed a slightly positive surface charge.

Through this screening, we identified several top-performing ionizable lipids with high selectivity (>90%) and robust protein expression levels for lung and spleen delivery. Among these, A3T2C7 (CP-LC-1495) exhibited remarkable lung selectivity (97.1%) and high protein expression (1.21 × 10^8^ p/s), reinforcing its potential for lung-targeted mRNA therapeutics. Our findings confirm that fine-tuning the hydrophobic chains and polar-head groups of ILs enables precise control over their biodistribution, ensuring targeted delivery to extrahepatic organs.

Furthermore, we examined the protein corona profiles of our most effective ILs to gain insights into their targeting mechanisms. Our data show that the lipid composition strongly influences their protein adsorption patterns, affecting their physicochemical properties and ultimately controlling tissue tropism. Lung-targeting LNPs exhibited distinct protein enrichment, including of vitronectin and prothrombin, supporting previously described mechanisms of protein corona-mediated lung selectivity [35]. In contrast, the protein-corona composition of spleen-targeting LNPs lacked a clear correlation with known targeting pathways, suggesting an alternative, but as-yet unidentified, mechanism of action.

In conclusion, these findings provide a strong foundation for the rational design of LNP systems with enhanced organ-targeting precision, guided by the structure–activity relationships of ionizable lipids and the LNP zeta potential. Understanding the molecular basis of extrahepatic targeting, including the key protein interactions that drive LNP accumulation in extrahepatic tissue, will be critical for optimizing these formulations for clinical use. Future studies should aim to elucidate these mechanisms and to evaluate the full safety profile, stability, and therapeutic potential of these LNPs in clinically relevant models. This approach holds significant promise for improving the specificity and efficacy of mRNA-based therapies, enabling more precise extrahepatic delivery and expanding the therapeutic landscape for a broad range of clinical applications, including in vaccines, cancer immunotherapy, and treatments for genetic and inflammatory diseases.

## Data Availability

Data is contained within the article or Supplementary Materials.

## Supplementary Materials

The following supporting information can be downloaded at: https://www.mdpi.com/article/10.3390/pharmaceutics17040459/s1, Supplementary Methods. Figure S1: Molecular structures of candidates to assess their potential for selective lung expression in used in Figure 1D; Figure S2: NMR and MS characterization of A3T2C7. Figure S3. Cell viability assays; Figure S4: HPLC-CAD data; Figure S5: LNP size distribution; Table S1: Compilation of the detected *m/z* values in the mass spectra of ionizable lipids; Table S2: Characterization of formulated LNPs by microfluidics; Table S3: Organ specificity (%) for LNPs containing ionizable lipids in Figure 2; Table S4: Organ specificity (%) for LNPs containing ionizable lipids in Figure 3; Table S5: Summary of top 30 most abundant proteins in the A1T2C3-Lung LNP protein corona; Table S6: Summary of top 30 most abundant proteins in the A3T2C7-Lung LNP protein corona; Table S7: Summary of top 30 most abundant proteins in the MC3-DOTAP-Lung LNP protein corona; Table S8: Summary of top 30 most abundant proteins in the A2T2C9-Spleen LNP protein corona; Table S9: Summary of top 30 most abundant proteins in the MC3-18:1 PA-Spleen LNP protein corona; Table S10: Summary of top 30 most abundant proteins in the SM-102 Liver LNP protein corona.
