# Supplementary material for: Predictive Lung- and Spleen-Targeted mRNA Delivery with Biodegradable Ionizable Lipids in Four-Component LNPs
Source: Pharmaceutics. 2025 Apr 2;17(4):459. doi: 10.3390/pharmaceutics17040459 (PMC12030499; doi:10.3390/pharmaceutics17040459)
Supplement: Supplementary file 1 [file pharmaceutics-17-00459-s001.zip › pharmaceutics-3549584-supplementary.pdf]

Predictive Lung and Spleen Targeted mRNA Delivery with Biodegradable Ionizable Lipids in Four-Component LNPs

Juan Heredero[‡,1] & Álvaro Peña[‡,1], Esther Broset[1], Beatriz Blandín[1], Diego de Miguel[1], Teresa Alejo[1], Alfonso Toro[1], Elena Mata[1], Alexandre López Gavín[1], Ana Gallego-Lleyda[1], Diego Casabona[1], Verónica Lampaya[1], Ana Larraga[1], Esther Pérez-Herrán[1], David Luna[1, 2, 3], Irene Orera[4], Eduardo Romanos[5], Alba García[5], Juan Martínez-Oliván[1,*] & Javier Giménez-Warren[1,*]

[1] Certest Pharma, Certest Biotec S. L., 50840, San Mateo de Gállego (Zaragoza), Spain

[2] Department of Theoretical Physics, Faculty of Science, University of Zaragoza, Pedro Cerbuna s/n, 50009, Zaragoza, Spain

[3] Institute for Biocomputation and Physics of Complex Systems (BIFI), University of Zaragoza, Mariano Esquillor s/n, 50018, Zaragoza, Spain

[4] Proteomics Research Core Facility, Aragon Health Sciences Institute (IACS), 50009 Zaragoza, Spain

[5] Medical Imaging and Phenotyping Core Facility, Aragon Health Sciences Institute (IACS), 50009 Zaragoza, Spain

‡ These authors contributed equally to this work.

* Authors to whom correspondence should be addressed.

Keywords: mRNA, ionizable lipids, lipid nanoparticles, extrahepatic delivery, lung targeting, spleen targeting, four-component LNPs, protein corona.

SUPLEMENTARY METHODS

General synthesis of thiolactone-sulfurs (Ty)

Synthesis T1:

Intermediate 1a

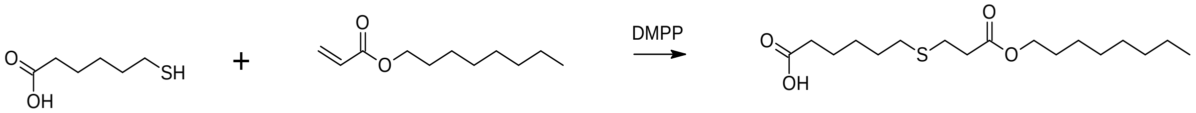

Intermediate 1a: 6-Mercaptohexanoic acid (2.8 mmol, 0.40 g) and n-octyl acrylate (4.2 mmol, 0.90 ml) were dissolved in 4 ml of ACN (anhydrous) at room temperature. Then, dimethylphosphine (0.70 mmol, 0.1 ml) was added as catalyst. The reaction mixture was stirred under argon atmosphere at room temperature 24h and was followed by HPLC-CAD-MS. Then, the reaction crude was evaporated under reduced pressure and was purified by flash chromatography (gradient of hexane (1% AcOH)/ethyl acetate (1%AcOH): 100/0 to 0/100) to afford intermediate 2b (98%). Intermediate 1a was characterized by HPLC-CAD-MS. MS (ES): experimental m/z $[M+Na]^+$ = 355.20; theorical m/z $[M+Na]^+$ =355.18.

T1

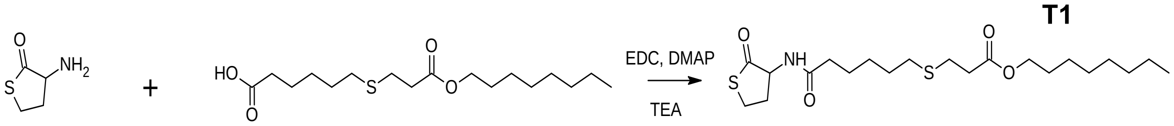

T1: DL-homocysteine thiolactone hydrochloride (3.2 mmol, 0.50 g) was dissolved in 30 mL of anhydrous dichloromethane at room temperature. Then triethylamine (3.2 mmol, 0.45 ml) was added followed by EDC hydrochloride (3.2 mmol, 0.63 g), 4-(dimethylamino)pyridine (0.49 mmol, 0.061 g) and intermediate 1a (2.5 mmol, 0.82 g). The reaction mixture was stirred at room temperature overnight under an argon atmosphere. Then, the reaction crude was washed once using distilled water (40 mL) and with saturated brine (40 mL). The organic layer was dried with anhydrous MgSO4, filtered, and evaporated under reduced pressure. The resulting residue was purified by flash chromatography (gradient of hexane/ethyl acetate: 100/0 to 0/100) to afford T1 (79%). T1 was characterized by HPLC-CAD-MS. MS (ES): experimental m/z $[M+Na]^+$ = 454.13; theorical m/z $[M+Na]^+$ =454.19.

Synthesis of T2,T3 and T4:

Intermediate 2a

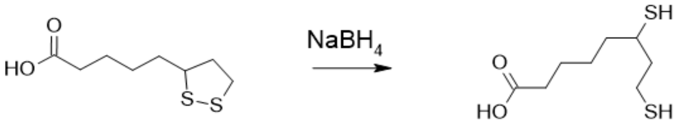

Intermediate 2a: Lipoic acid (2.0 g, 10.3 mmol) is dissolved in 50 ml NaHCO3 (aq, 0.25M). Subsequently, NaBH4 is added in small portions at 0ºC. The solution changes from yellow to colorless. The reaction mixture was stirred at 0ºC for 2 hours. Then, the reaction crude is quenched with HCL (37%) until pH=1. Afterwards, the crude was extracted with toluene three times. The organic layer was dried with anhydrous Na2SO4, filtered, and evaporated under reduced pressure to afford intermediate compound Intermediate 2a (quantitative yield). Intermediate 2a was used without any further purification in the next step.

Intermediate 2b

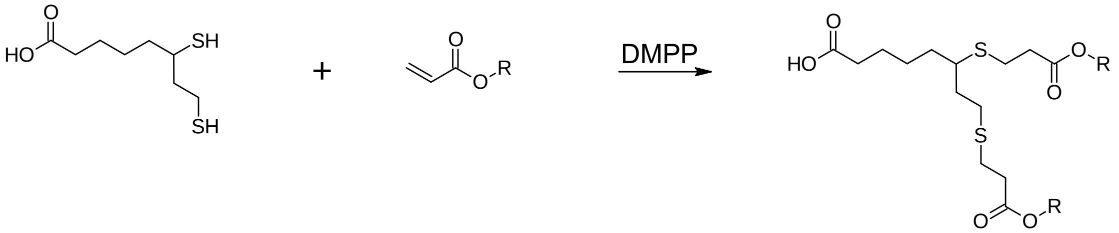

Intermediate 2b: Intermediate 2a (8.8 mmol) and the respective acrylate (n-octyl acrylate for T2; isooctyl acrylate for T3; 2-ethylhexylacrylate for T3) (26.3 mmol) were dissolved in 18 ml of ACN (anhydrous) at room temperature. Then, dimethylphosphine (3.3 mmol, 0.5 ml) was added as catalyst. The reaction mixture was stirred at room temperature 24h under an argon atmosphere and was followed by HPLC-CAD-MS. Then, the reaction crude was evaporated under reduced pressure and was purified by flash chromatography (gradient of hexane (1% AcOH)/ethyl acetate (1%AcOH): 100/0 to 0/100) to afford intermediate 2b. Intermediate 2b was characterized by HPLC-CAD-MS.

Intermediates 2c (T2, T3 or T4)

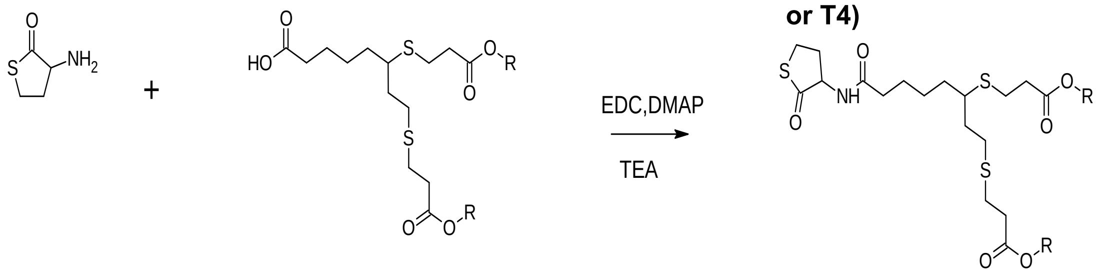

Intermediate 2c: DL-homocysteine thiolactone hydrochloride (1.3 g, 8.6 mmol) was dissolved in 75 mL of anhydrous dichloromethane at room temperature. Then triethylamine (1.2 ml, 8.6 mmol) was added followed by EDC hydrochloride (1.7 g, 8.6 mmol), 4-(dimethylamino)pyridine (0.2 mmol, 0.2 g) and intermediate 2c (6.6 mmol). The reaction mixture was stirred at room temperature overnight under an argon atmosphere. Then, the reaction crude was washed two times using distilled water (2 x 40 mL) and with saturated brine (40 mL). The organic layer was dried with anhydrous MgSO4, filtered, and evaporated under reduced pressure. The resulting residue was purified by flash chromatography (gradient of hexane/ethyl acetate: 100/0 to 0/100) to afford intermediates 2c, known as T2, T3 or T4. Intermediates 2c was characterized by HPLC-CAD-MS.

T2: MS (ES): experimental m/z $[M+H]^+$ = 676.30; theorical m/z $[M+H]^+$ =676.37.

T3: MS (ES): experimental m/z $[M+H]^+$ = 676.30; theorical m/z $[M+H]^+$ =676.37.

T4: MS (ES): experimental m/z $[M+H]^+$ = 676.30; theorical m/z $[M+H]^+$ =676.37.

Nuclear magnetic resonance (NMR)

NMR spectra was obtained using a Bruker Avance 400 MHz device and deuterated solvents (CDCl3, Sigma Aldrich). CDCl3 spectra contains approximately 0.03% of tetramethyl silane. (TMS).

Cytotox evaluation

HepG2 cell line was purchased from ATCC (cat. HB-8065) and A549 and Jurkat cells lines were purchased from Leibniz Institute (cat. ACC 107 and ACC 282). Cells were maintained in RPMI 1640 (Gibco, 31870074) for HepG2 and Jurkat or high-glucose Dulbecco's Modified Eagle's Medium (Merck, D6429) for A549, both supplemented with 10% fetal bovine serum (Sigma, F7524) 1% Penicillin-Streptomycin Solution (Gibco, 15140122) and 2 mM Glutamax (Fisher, 35050038) at 37 °C in a humidified incubator with 5% CO2. Cells were plated in 96-well plates at a density of 10.000 cells/well and incubated overnight. The corresponding LNPs encapsulating FLuc mRNA were added at 40 ng of mRNA per well. Media alone was employed as a negative control and doxorubicin was used as a positive control. After 24 h of incubation, 10 ul/well of PrestoBlue Cell Viability Reagent (Thermofisher) was added to each well and incubated for 1 h at 37 ºC and 5% CO2. PestoBlue-derived fluorescence was quantified using a FLUOstar Omega plate reader (BMG Labtech) and viability was determined by dividing the fluorescence of each well by the average fluorescence signal of the negative controls. Cell viability percentage was reported as mean ± SD of n = 3 technical replicates.

HPLC Method

HPLC-CAD chromatograms were obtained using a Vanquish HPLC equipped with Vanquish Charged Aerosol Detector (CAD)and a X Bridge – Waters - BEH C8 (4.6 mm, 50 mm, 2.5 µm) column at 65 °C. The detection was performed via Charged Aerosol Detector (CAD) plus simple quadrupole mass detector in positive mode (ISQ EC Single Quadrupole Mass Spectrometer (ThermoFisher Scientific)).

Gradient: (A: Water with 0.01% TFA; B: Acetonitrile with 0.01% TFA). The gradient was programmed as follows: 5% B at 0 min with a flow rate of 1 mL/min, held constant at 5% B until 0.5 min, then increasing linearly to 50% B from 0.5 to 1.5 min, followed by a further increase to 95% B from 1.5 to 11.5 min, maintained at 95% B from 11.5 to 12 min, then decreasing to 5% B from 12 to 12.5 min, and held at 5% B from 12.5 to 13 min. The flow rate remained constant at 1 mL/min throughout the run.

TABLES AND SUPPORTING FIGURES

CP-LC-0263

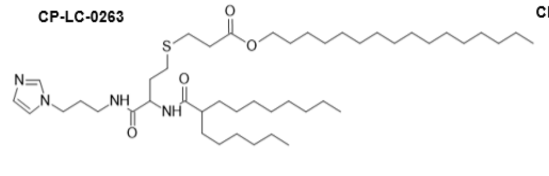

CP-LC-0474

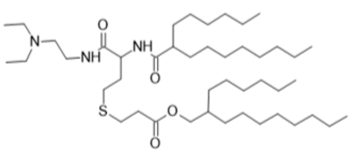

CP-LC-0729

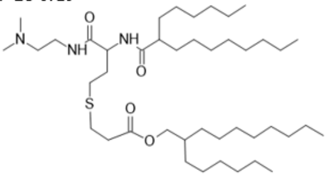

CP-LC-0733

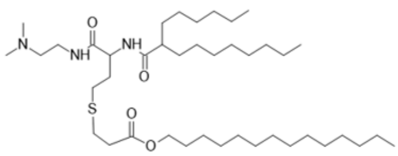

CP-LC-0743

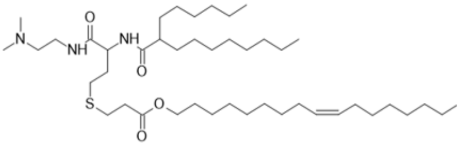

CP-LC-0797

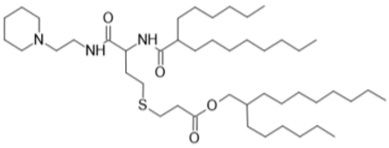

CP-LC-0811

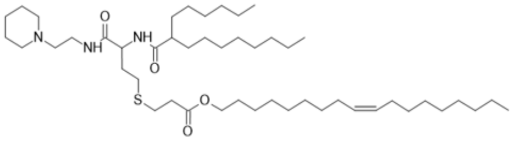

CP-LC-0846

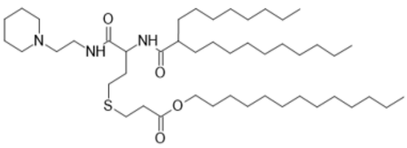

CP-LC-0847

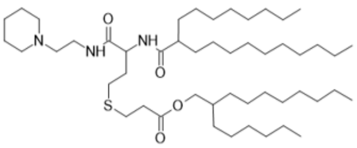

CP-LC-0850

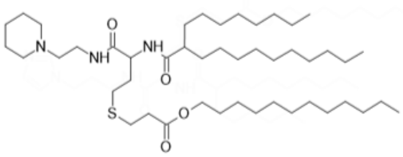

CP-LC-0866

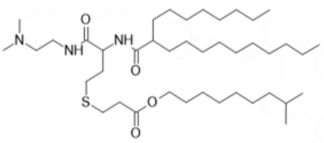

CP-LC-0878

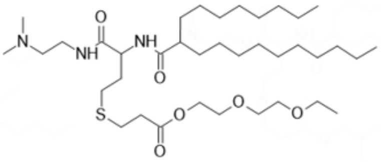

CP-LC-0879

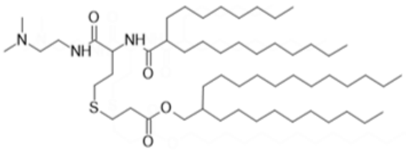

CP-LC-0958

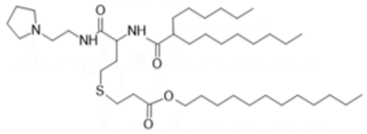

CP-LC-0977

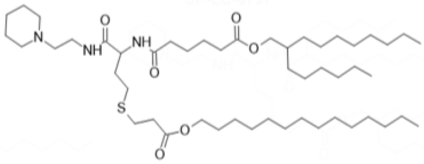

CP-LC-0988

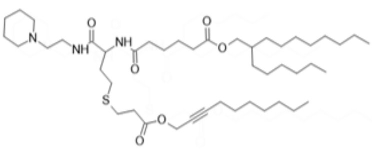

CP-LC-1008

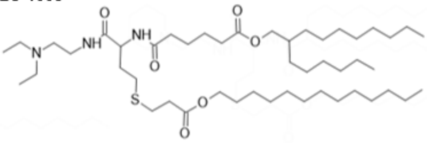

CP-LC-1065

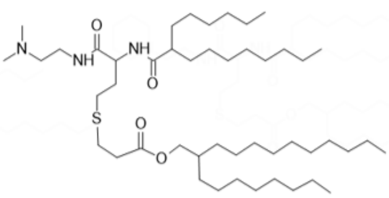

CP-LC-1068

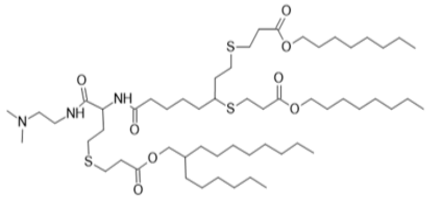

CP-LC-1069

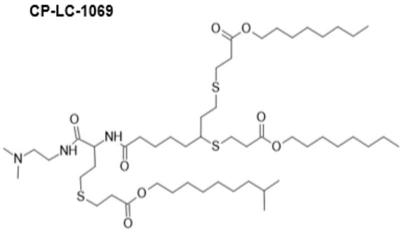

CP-LC-1072

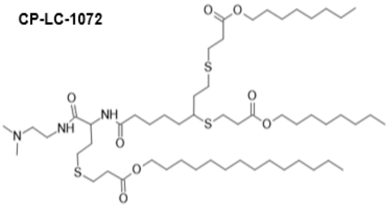

CP-LC-1078

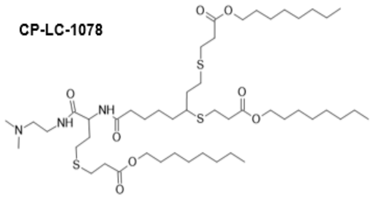

CP-LC-1079

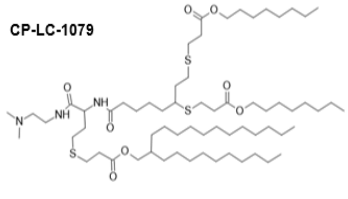

CP-LC-1101

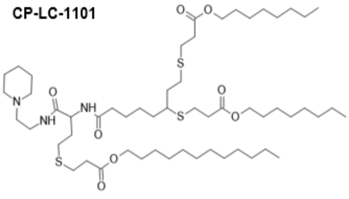

CP-LC-1104

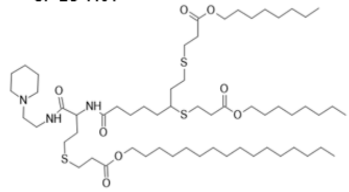

CP-LC-1107

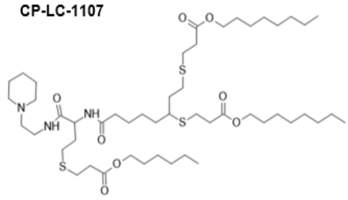

CP-LC-1125

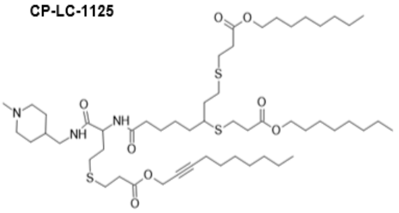

CP-LC-1134

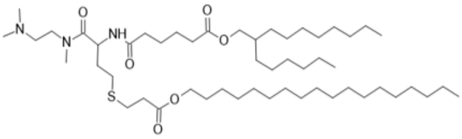

CP-LC-1135

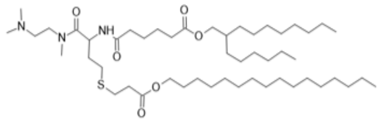

CP-LC-1146

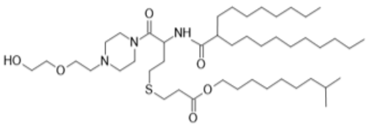

CP-LC-1218

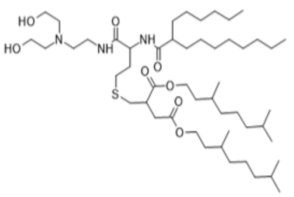

CP-LC-1221

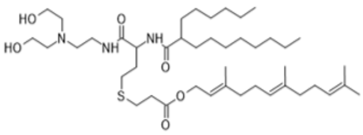

CP-LC-1224

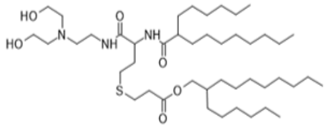

CP-LC-1229

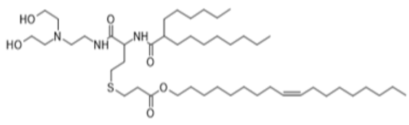

CP-LC-1230

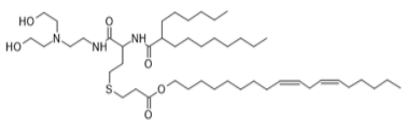

Figure S1. Molecular structures of candidates to assess their potential for selective lung expression in used in Figure 1D.

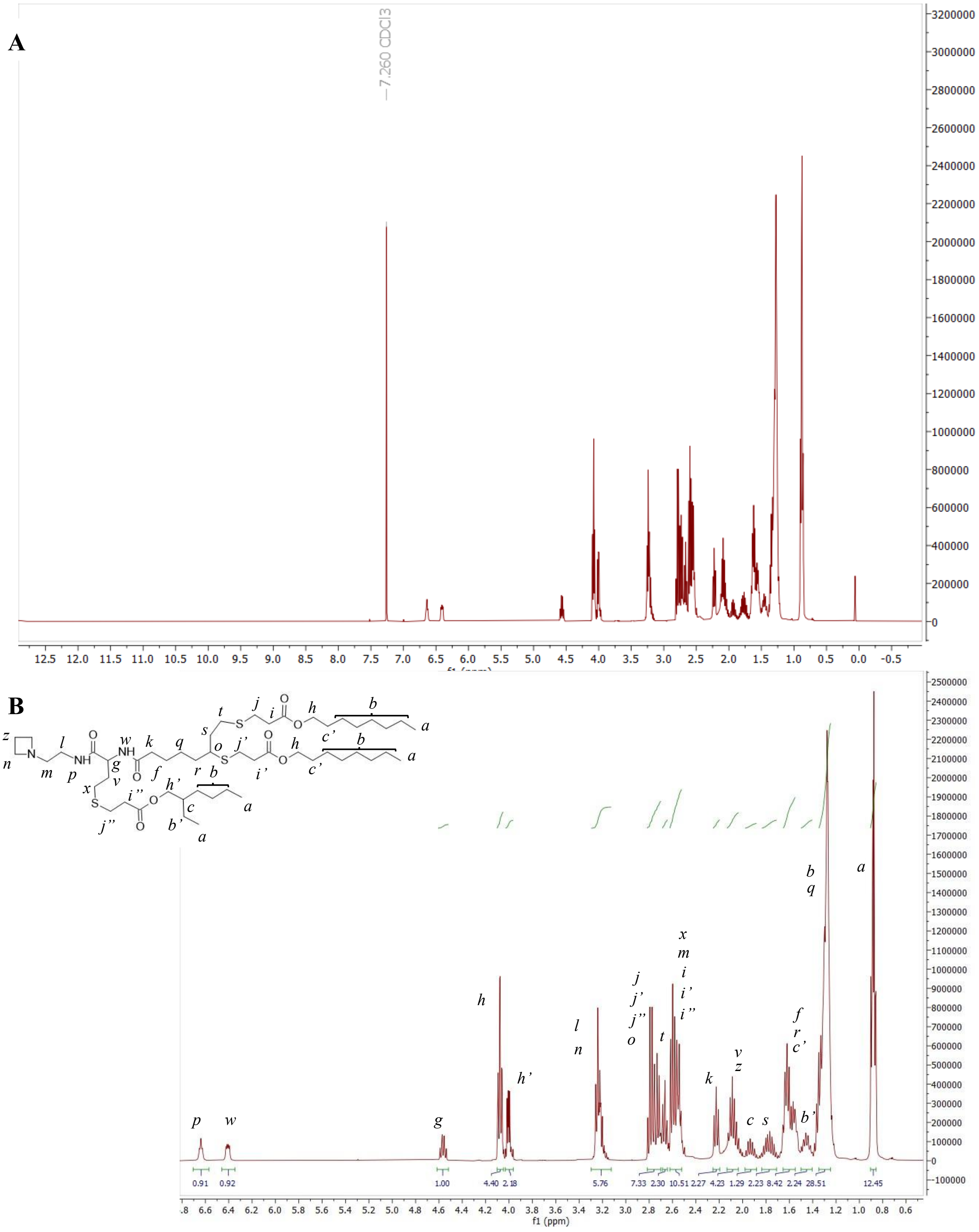
A
—7.260 CDCl3
B
f1 (ppm)
p
w
g
h
h'
l
n
j
j'
j''
o
t
x
m
i
i'
i''
k
v
z
c
s
f
r
c'
b'
b
q
a
0.91
0.92
1.00
4.40
2.18
5.76
7.33
2.30
10.51
2.27
4.23
1.29
2.23
8.42
2.24
28.51
12.45

C

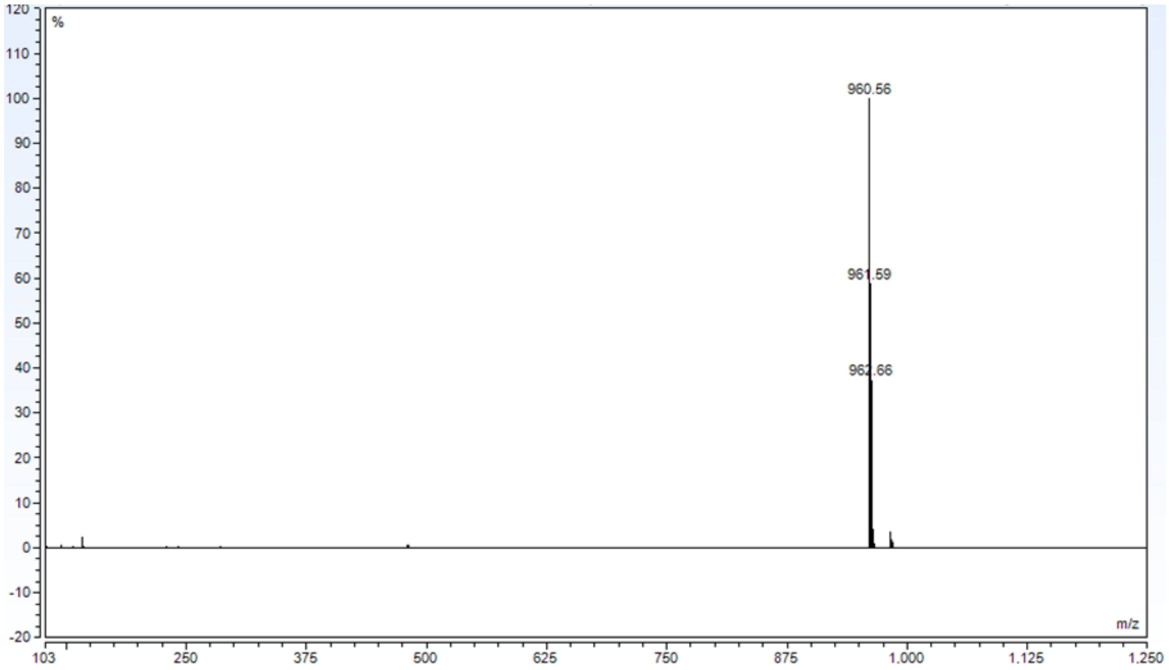

Figure S2. **NMR and MS characterization of A3T2C7.** A) Complete ^{1}H-NMR spectra of A3T2C7 (CP-LC-1495). **B)** Extended ^{1}H-NMR spectra of A3T2C7 (CP-LC-1495) with peak assignments. **C)** Mass spectrum of A3T2C7 (CP-LC-1495).

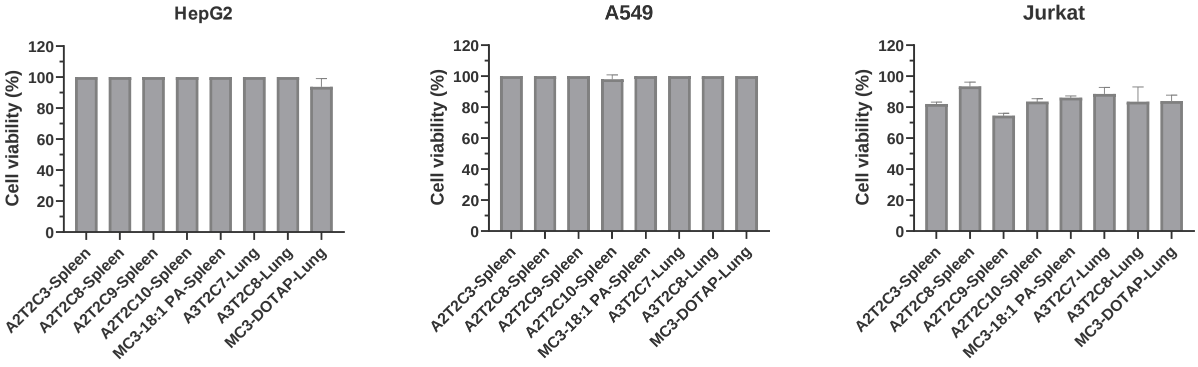

Figure S3. Cell viability assays. Cell viability of HepG2, A549 and Jurkat cells 24 hours after transfection with LNPs containing 40 ng of mRNA. Data are presented as mean ± SD.

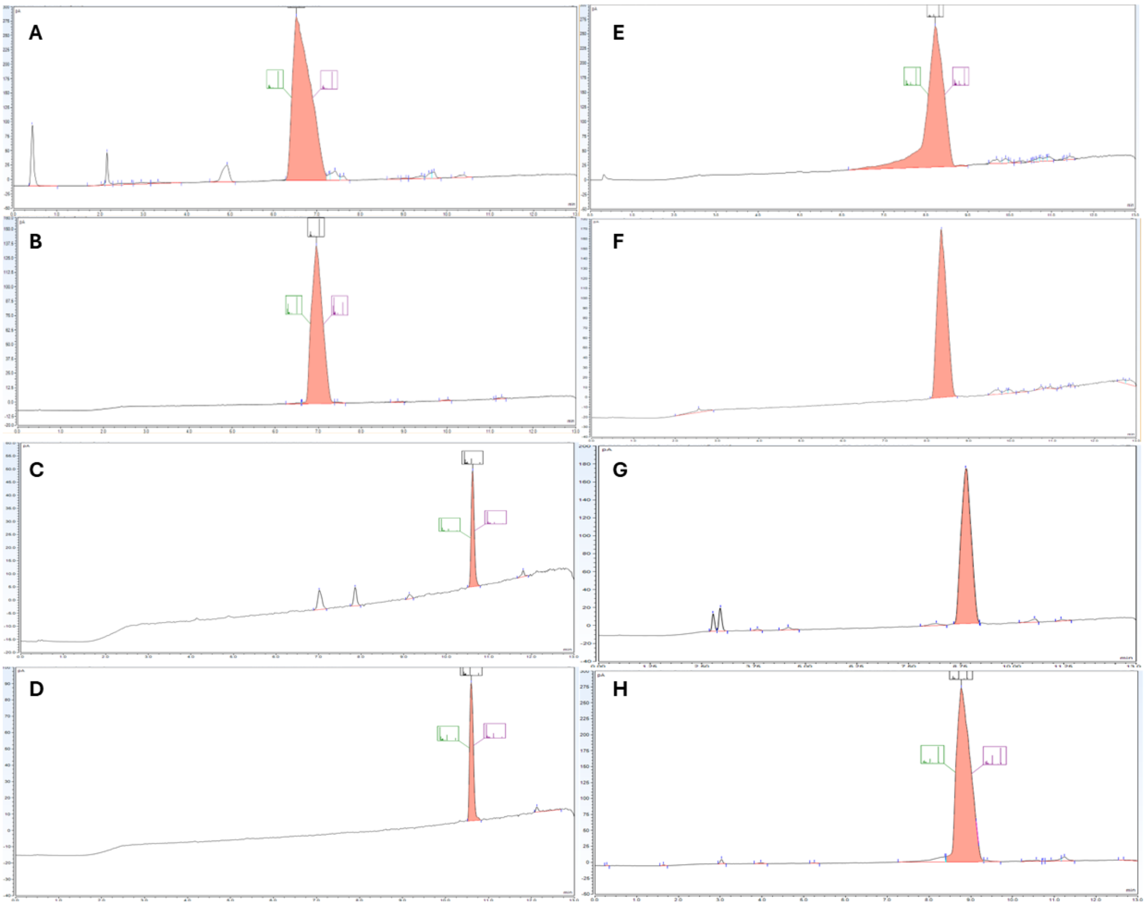

Figure S4. HPLC-CAD data. A) HPLC-CAD chromatogram of crude A3T2C7 (CP-LC-1495), with the target product highlighted in red. **B)** HPLC-CAD chromatogram of pure A3T2C7 (CP-LC-1495), with the target product highlighted in red (Pure compound: 97%). **C)** HPLC-CAD chromatogram of crude A2T2C10, with the target product highlighted in red. **D)** HPLC-CAD chromatogram of pure A2T2C10, with the target product highlighted in red. (Pure compound: 96%). **E)** HPLC-CAD chromatogram of crude A2T2C3, with the target product highlighted in red. **F)** HPLC-CAD chromatogram of pure A2T2C3, with the target product highlighted in red. (Pure compound: 94%). **G)** HPLC-CAD chromatogram of crude A2T2C9, with the target product highlighted in red. **H)** HPLC-CAD chromatogram of pure A2T2C9, with the target product highlighted in red. (Pure compound: 94%).

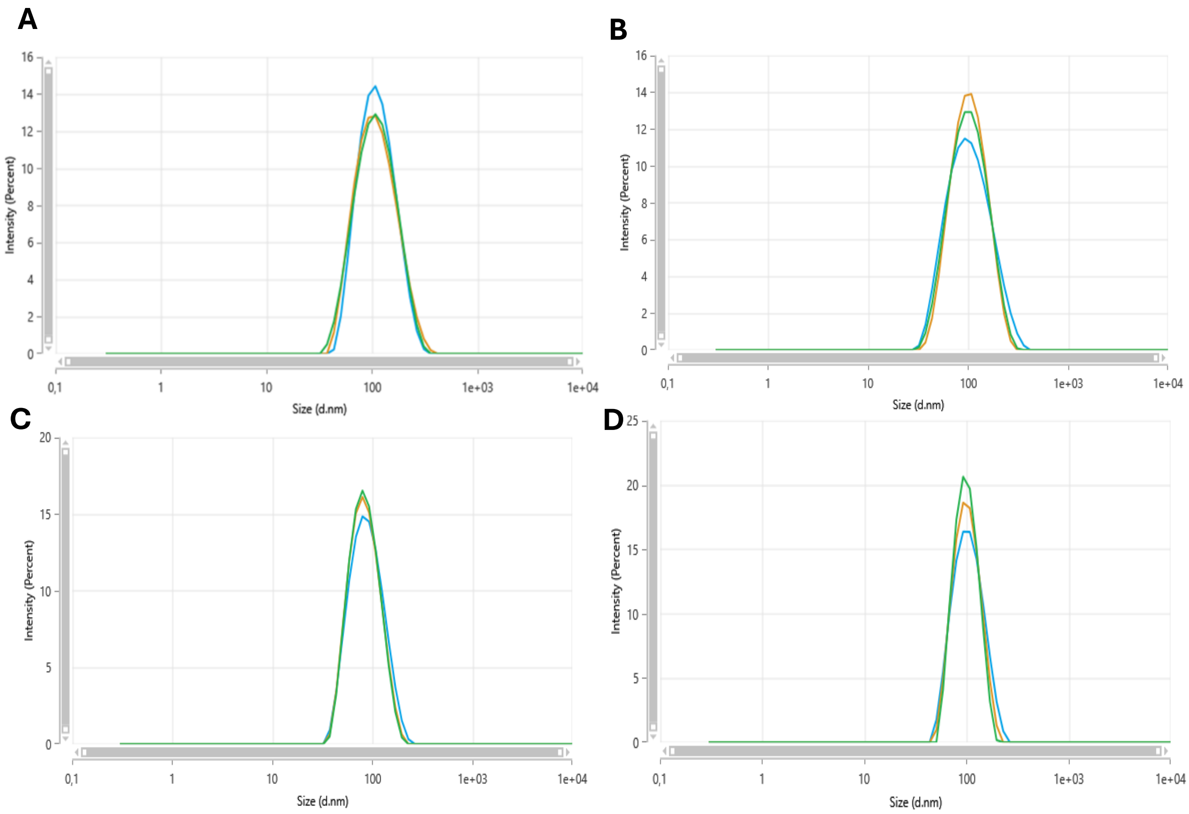

Figure S5. LNP size distribution. Size distribution of lipid nanoparticles (LNPs) measured by Dynamic Light Scattering (three replicates for each plot). **A)** Lipid nanoparticle containing A2T2C10 as ionizable lipid. **B)** Lipid nanoparticle containing A2T2C3 as ionizable lipid. **C)** Lipid nanoparticle containing A1T2C3 as ionizable lipid. **D)** Lipid nanoparticle containing A3T2C7 as ionizable lipid.

Table S1. Compilation of the detected m/z values in the mass spectra of ionizable lipids

Ionizable Lipid	Theoretical [M+H]+	Experimental [M+H]+
A1T1C1	816.59	816.62
A1T1C2	788.56	788.53
A1T1C3	774.54	774.51
A1T1C4	760.53	760.45
A1T1C5	704.47	704.48
A1T1C6	704.47	704.48
A1T1C7	704.47	704.48
A1T1C8	760.53	760.65
A1T1C9	816.59	816.54
A1T1C10	928.72	928.68
A1T1C11	842.61	842.68
A1T2C1	1060.74	1060.7
A1T2C2	1032.71	1032.96
A1T2C3	1018.69	1018.87

A1T2C4	1004.68	1004.73
A1T2C5	948.62	948.92
A1T2C6	948.62	948.88
A1T2C7	948.61	948.84
A1T2C8	1004.68	1004.65
A1T2C9	1060.74	1060.98
A1T2C10	1172.87	1173.09
A1T2C11	1086.76	1086.98
A1T3C1	1060.74	1061.02
A1T3C2	1032.63	1032.98
A1T3C3	1018.7	1018.95
A1T3C4	1004.68	1004.85
A1T3C5	948.62	948.61
A1T3C6	948.62	948.63
A1T3C7	948.62	948.63
A1T3C8	1004.69	1004.67
A1T3C9	1060.75	1061.05
A1T3C10	1172.87	1173.03
A1T3C11	1086.76	1086.99
A1T4C1	1060.74	1060.99
A1T4C2	1032.71	1032.73
A1T4C3	1018.7	1018.72
A1T4C4	1004.68	1004.65
A1T4C5	948.62	948.64
A1T4C6	948.62	948.66
A1T4C7	948.62	948.67
A1T4C8	1004.68	1004.68
A1T4C9	976.65	976.7
A1T4C10	1172.87	1173.9
A1T4C11	1086.76	1086.83
A2T2C1	1083.72	1083.76
A2T2C2	1055.69	1056.06
A2T2C3	1041.68	1041.77
A2T2C4	1027.66	1027.63
A2T2C5	971.6	971.65
A2T2C6	971.6	971.67
A2T2C7	971.6	971.56
A2T2C8	1027.66	1027.69
A2T2C9	1083.68	1083.7
A2T2C10	1195.89	1196.09
A2T2C11	1109.72	1109.78
A3T2C1	1072.74	1072.99
A3T2C2	1044.71	1044.74
A3T2C3	1030.7	1030.66
A3T2C4	1016.68	1016.67
A3T2C5	960.62	960.65
A3T2C6	960.62	960.96
A3T2C7	960.62	960.56
A3T2C8	1016.68	1016.78
A3T2C9	1072.74	1072.71

A3T2C10	1184.87	1185.13
A3T2C11	1098.76	1098.77

Table S2. Characterization of formulated LNPs by microfluidics.

	Particle Size(nm)	P.D.I.	Zeta Potential (mV)	%EE
A1T1C1	X	X	X	X
A1T1C2	70.1	0.28	6.5	92.7
A1T1C3	69.4	0.25	6.2	91.6
A1T1C4	68.6	0.13	3.5	92.3
A1T1C5	90.6	0.25	1.5	96.2
A1T1C6	124.1	0.19	5.4	95.8
A1T1C7	160.7	0.10	2.5	95.6
A1T1C8	97.1	0.18	7.2	91.3
A1T1C9	87.8	0.13	3.0	92.9
A1T1C10	112.9	0.24	0.5	95.8
A1T1C11	89.7	0.23	10.6	93.0
A1T2C1	89.8	0.16	3.9	98.5
A1T2C2	94.8	0.24	2.2	99.9
A1T2C3	79.8	0.12	7.0	94.3
A1T2C4	90.1	0.21	1.1	99.9
A1T2C5	129.8	0.13	7.3	98.3
A1T2C6	102.1	0.13	1.7	99.9
A1T2C7	86.7	0.14	2.4	100.0
A1T2C8	87.7	0.06	-1.0	94.6
A1T2C9	103.0	0.25	-1.2	100.0
A1T2C10	130.8	0.06	3.7	96.2
A1T2C11	97.8	0.10	2.1	100.0
A1T3C1	97.0	0.21	2.9	95.7
A1T3C2	90.6	0.16	-1.1	94.8
A1T3C3	96.1	0.08	-0.1	97.6
A1T3C4	88.3	0.08	0.7	94.1
A1T3C5	103.0	0.12	1.4	94.0
A1T3C6	105.5	0.06	-2.9	94.5
A1T3C7	92.8	0.09	-0.1	96.8
A1T3C8	95.9	0.22	-5.4	96.2
A1T3C9	90.9	0.16	-2.8	97.0
A1T3C10	92.1	0.09	-3.5	94.6
A1T3C11	98.2	0.12	0.5	96.4
A1T4C1	X	X	X	X
A1T4C2	107.1	0.16	5.0	91.7
A1T4C3	110.9	0.21	2.7	91.7
A1T4C4	X	X	X	X
A1T4C5	108.2	0.09	1.2	91.9
A1T4C6	119.6	0.18	1.4	91.9
A1T4C7	140.8	0.20	-0.5	90.7
A1T4C8	101.2	0.10	-2.4	92.0
A1T4C9	109.4	0.15	-1.6	92.8
A1T4C10	105.3	0.14	0.7	92.5
A1T4C11	114.6	0.19	6.3	92.4

A2T2C1	69.3	0.13	-13.3	90.0
A2T2C2	83.3	0.29	-19.1	89.6
A2T2C3	91.8	0.23	-7.9	92.2
A2T2C4	65.4	0.12	-13.1	91.1
A2T2C5	81.6	0.27	-9.7	88.7
A2T2C6	75.2	0.17	-11.4	88.7
A2T2C7	78.0	0.21	-11.0	89.4
A2T2C8	75.3	0.26	-11.0	92.3
A2T2C9	86.2	0.18	-11.3	91.3
A2T2C10	101.5	0.15	-13.2	88.8
A2T2C11	78.2	0.23	-12.6	89.9
A3T2C1	148.3	0.23	9.7	97.3
A3T2C2	108.5	0.16	12.4	97.9
A3T2C3	99.4	0.19	9.7	98.2
A3T2C4	149.8	0.27	9.2	97.8
A3T2C5	133.1	0.29	6.4	96.5
A3T2C6	143.0	0.26	5.7	96.9
A3T2C7	96.2	0.08	5.8	97.2
A3T2C8	100.1	0.10	3.0	97.0
A3T2C9	123.2	0.26	1.6	98.3
A3T2C10	126.4	0.28	9.7	95.3
A3T2C11	104.4	0.10	3.3	95.4

LNPs were formulated by microfluidic mixing. The resulting lipid particles encapsulating mRNAs were dialyzed overnight against a pH 8 Tris buffer solution containing 15% sucrose. Lipid particles were characterized by measuring their size distribution, polydispersity and Z-potential by dynamic light scattering (DLS). mRNA encapsulation was assessed by Quant-IT® Ribogreen following the manufacturer’s instructions. A1T1C1, A1T4C1 and A1T4C4 LNPs precipitated and were not characterized.

Table S3. Organ specificity (%) for LNPs containing ionizable lipids in Figure 2. Mice were i.v injected with mRNA-Luc-loaded LNPs at an mRNA dose of 0.5 mg/kg (n= 2 biologically independent samples). Data are presented as mean values.

Ionizable lipid	%Liver	%Kidneys	%Lung	%Heart	%Intestine	%Spleen
A1T1C1	X	X	X	X	X	X
A1T1C2	22.55	5.25	26.10	9.79	6.84	29.47
A1T1C3	30.52	6.54	7.03	9.85	12.10	33.96
A1T1C4	X	X	X	X	X	X
A1T1C5	37.25	8.73	6.99	12.77	19.15	15.11
A1T1C6	15.10	10.96	12.88	19.78	16.75	24.53
A1T1C7	13.58	13.35	13.76	25.67	16.16	17.48
A1T1C8	3.43	4.00	68.78	6.23	5.20	12.37
A1T1C9	6.96	1.76	29.88	3.89	4.43	53.07
A1T1C10	44.19	3.75	13.62	6.14	4.46	27.85
A1T1C11	23.91	5.43	41.78	6.62	4.85	17.41
A1T2C1	3.80	1.62	83.93	2.40	3.89	4.36
A1T2C2	14.89	4.51	46.87	7.39	12.47	13.88
A1T2C3	0.95	0.29	94.06	0.50	1.65	2.55
A1T2C4	15.92	3.13	54.27	5.42	8.40	12.86
A1T2C5	53.45	0.94	41.61	0.51	1.01	2.47
A1T2C6	6.56	2.19	66.62	3.27	5.45	15.91
A1T2C7	14.30	6.13	51.54	7.91	8.02	13.10
A1T2C8	24.49	4.33	18.03	6.09	8.05	39.00
A1T2C9	29.84	4.72	15.96	6.01	8.32	35.14
A1T2C10	3.97	1.59	33.28	2.58	6.42	52.16
A1T2C11	7.32	3.42	65.16	5.05	7.36	11.70
A1T3C1	4.64	1.85	60.69	3.01	3.65	26.15
A1T3C2	12.18	2.15	38.27	2.76	6.64	38.01
A1T3C3	1.79	0.44	83.97	0.80	2.50	10.49
A1T3C4	22.12	1.10	25.33	2.34	2.30	46.80
A1T3C5	7.86	1.50	52.62	1.78	5.18	31.06
A1T3C6	93.02	0.32	0.78	0.49	0.48	4.91
A1T3C7	3.84	1.48	75.59	2.48	2.60	14.02
A1T3C8	63.78	1.19	2.14	2.27	4.33	26.29
A1T3C9	12.16	0.63	7.30	0.71	1.72	77.48
A1T3C10	30.82	0.71	7.62	0.96	1.03	58.87
A1T3C11	5.02	2.31	55.72	3.17	4.90	28.89
A1T4C1	X	X	X	X	X	X
A1T4C2	1.92	0.51	63.57	0.55	1.67	31.79
A1T4C3	7.83	0.72	36.66	1.03	1.76	51.99
A1T4C4	2.43	0.77	47.14	0.97	2.04	46.64
A1T4C5	4.17	1.42	46.07	1.71	2.42	44.21
A1T4C6	4.47	1.13	62.52	1.11	1.97	28.80
A1T4C7	12.96	1.05	38.15	0.94	3.90	43.00
A1T4C8	14.41	0.42	16.60	0.66	1.58	66.33
A1T4C9	26.25	1.57	24.30	1.70	2.30	43.88
A1T4C10	23.66	0.95	28.43	1.01	1.92	44.03
A1T4C11	3.28	0.44	63.62	0.46	1.42	30.78

Table S4. Organ specificity (%) for LNPs containing ionizable lipids in Figure 3. Mice were i.v injected with mRNA-Luc-loaded LNPs at an mRNA dose of 0.5 mg/kg (n= 2 biologically independent samples). Data are presented as mean values.

Ionizable lipid	%Liver	%Kidneys	%Lung	%Heart	%Intestine	%Spleen
A2T2C1	8.87	4.49	2.23	5.66	5.57	73.19
A2T2C2	8.56	1.60	1.03	2.52	2.50	83.79
A2T2C3	21.59	0.61	2.94	0.86	1.07	72.93
A2T2C4	15.89	3.33	2.78	6.66	5.81	65.52
A2T2C5	9.99	2.63	1.72	5.52	4.45	75.69
A2T2C6	16.84	2.49	4.87	4.44	3.71	67.65
A2T2C7	11.89	2.79	4.93	4.23	3.32	72.84
A2T2C8	9.27	2.21	3.24	3.09	2.09	80.10
A2T2C9	2.64	0.22	0.47	0.27	0.85	95.56
A2T2C10	9.56	1.04	1.80	1.66	1.49	84.46
A2T2C11	23.13	2.22	1.57	2.88	2.89	67.30
A3T2C1	2.12	1.77	83.36	2.97	2.99	6.79
A3T2C2	0.51	0.60	96.04	0.74	0.95	1.16
A3T2C3	2.21	1.47	87.29	3.32	2.61	3.09
A3T2C4	0.38	0.28	97.55	0.36	0.71	0.72
A3T2C5	0.61	0.37	95.20	0.58	1.16	2.09
A3T2C6	0.91	0.81	77.46	1.50	1.56	17.76
A3T2C7	0.65	0.25	97.08	0.17	0.61	1.27
A3T2C8	0.55	0.35	95.90	0.53	0.69	1.98
A3T2C9	12.33	0.57	71.60	1.23	1.75	12.52
A3T2C10	55.57	2.98	5.25	5.66	3.48	27.07
A3T2C11	2.24	1.28	60.02	1.47	2.05	32.94

Table S5 Summary of top 30 most abundant proteins in the A1T2C3-Lung LNP protein corona.

**Protein Name**	**Abundance (NSAF)**
Actin, cytoplasmic 1	0.981333333
Vitronectin	0.669456067
Actin, alpha cardiac muscle 1	0.580901857
Prothrombin	0.573954984
Clusterin	0.53674833
Fibrinogen beta chain	0.519348269
Apolipoprotein E	0.517350158
Serum paraoxonase/arylesterase 1	0.504225352
Fibrinogen gamma chain	0.485651214
Immunoglobulin kappa constant	0.476635514
Apolipoprotein A-IV	0.46969697
Complement C1s subcomponent	0.462209302
Albumin	0.449917898
Hemoglobin subunit beta	0.421768707
Tubulin beta-1 chain	0.414634146
Apolipoprotein A-I	0.411985019
C4b-binding protein alpha chain	0.400335008
Hemoglobin subunit alpha	0.330985915
Fibrinogen alpha chain	0.308314088
Profilin-1	0.3
Tubulin beta chain	0.295045045
Alpha-actinin-1	0.289237668
Tubulin beta-4B chain	0.283146067
Tubulin alpha-1B chain	0.279379157
Tubulin alpha-4A chain	0.276785714
Vitamin K-dependent protein S	0.275147929
Complement C4-B	0.274655963
Complement C4-A	0.273509174
14-3-3 protein zeta/delta	0.273469388
Hyaluronan-binding protein 2	0.269642857

Table S6. Summary of top 30 most abundant proteins in the A3T2C7-Lung LNP protein corona

**Protein Name**	**Abundance (NSAF)**
Apolipoprotein A-IV	0.97979798
Actin, cytoplasmic 1	0.925333333
Vitronectin	0.69874477
Fibrinogen beta chain	0.619144603
Actin, alpha cardiac muscle 1	0.564986737
Apolipoprotein E	0.558359621
Immunoglobulin kappa constant	0.551401869
Clusterin	0.534521158
Albumin	0.50410509
Prothrombin	0.501607717
Hemoglobin subunit beta	0.496598639
Fibrinogen gamma chain	0.485651214
Apolipoprotein A-I	0.483146067
Serum paraoxonase/arylesterase 1	0.473239437
Complement C1s subcomponent	0.447674419
Tubulin beta-1 chain	0.416851441
C4b-binding protein alpha chain	0.351758794
14-3-3 protein zeta/delta	0.346938776
Hemoglobin subunit alpha	0.345070423
Fibrinogen alpha chain	0.344110855
Hemoglobin subunit delta	0.319727891
Tubulin alpha-1B chain	0.317073171
Tubulin alpha-4A chain	0.314732143
Alpha-actinin-1	0.307174888
Tubulin alpha-1A chain	0.305986696
Immunoglobulin heavy constant gamma 1	0.30075188
Tubulin alpha-1C chain	0.293986637
Tropomyosin alpha-4 chain	0.286290323
Tubulin beta chain	0.283783784
Complement C4-B	0.279816514

Table S7. Summary of top 30 most abundant proteins in the MC3-DOTAP-Lung LNP protein corona

Protein Name	Abundance (NSAF)
Fibrinogen beta chain	1.152749491
Fibrinogen gamma chain	0.969094923
Fibrinogen alpha chain	0.616628176
Actin, cytoplasmic 1	0.557333333
Apolipoprotein A-IV	0.507575758
Prothrombin	0.495176849
Albumin	0.461412151
Actin, alpha cardiac muscle 1	0.350132626
Apolipoprotein A-I	0.314606742
Keratin, type I cytoskeletal 10	0.297945205
Keratin, type II cytoskeletal 1	0.287267081
Vitronectin	0.286610879
C4b-binding protein alpha chain	0.281407035
Alpha-1-antitrypsin	0.265550239
Immunoglobulin kappa constant	0.242990654
Inter-alpha-trypsin inhibitor heavy chain H2	0.233615222
Hemoglobin subunit beta	0.217687075
Serum paraoxonase/arylesterase 1	0.216901408
Immunoglobulin heavy constant gamma 1	0.20802005
Clusterin	0.198218263
Heparin cofactor 2	0.196392786
Protein AMBP	0.196022727
Inter-alpha-trypsin inhibitor heavy chain H1	0.190998902
Complement C1r subcomponent	0.187234043
Keratin, type II cytoskeletal 2 epidermal	0.186228482
Plasma protease C1 inhibitor	0.184
Complement C1s subcomponent	0.167151163
Tubulin beta-1 chain	0.166297118
Vitamin K-dependent protein S	0.162721893
Keratin, type I cytoskeletal 9	0.149277689

Table S8. Summary of top 30 most abundant proteins in the A2T2C9-Spleen LNP protein corona.

**Protein Name**	**Abundance (NSAF)**
Actin, cytoplasmic 1	1.202666667
Actin, alpha skeletal muscle	0.710875332
Hemoglobin subunit beta	0.489795918
Albumin	0.481116585
Tubulin beta-1 chain	0.467849224
Alpha-actinin-1	0.396860987
Immunoglobulin kappa constant	0.38317757
Fermitin family homolog 3	0.35832084
Tubulin alpha-4A chain	0.345982143
Tubulin alpha-1B chain	0.328159645
Myosin-9	0.321428571
14-3-3 protein zeta/delta	0.318367347
Hemoglobin subunit alpha	0.316901408
Glyceraldehyde-3-phosphate dehydrogenase	0.31641791
Pyruvate kinase PKM	0.316384181
Tubulin beta chain	0.315315315
Vinculin	0.312169312
Ras-related protein Rap-1b	0.304347826
Tubulin alpha-1A chain	0.303769401
Profilin-1	0.3
Tubulin beta-4B chain	0.298876404
Tubulin alpha-1C chain	0.296213808
Apolipoprotein A-IV	0.295454545
Fibrinogen beta chain	0.295315682
Talin-1	0.293191657
Pleckstrin	0.282857143
Transgelin-2	0.281407035
Hemoglobin subunit delta	0.272108844
Cofilin-1	0.271084337
Tubulin alpha-3C chain	0.262222222

Table S9. Summary of top 30 most abundant proteins in the MC3-18:1 PA-Spleen LNP protein corona

Protein Name	Abundance (NSAF)
Actin, cytoplasmic 1	1.549333333
Actin, alpha cardiac muscle 1	0.848806366
Actin, alpha skeletal muscle	0.848806366
Albumin	0.642036125
Hemoglobin subunit beta	0.639455782
Tubulin beta-1 chain	0.638580931
Alpha-actinin-1	0.516816143
Ras-related protein Rap-1b	0.516304348
Immunoglobulin kappa constant	0.514018692
Fermitin family homolog 3	0.460269865
Tubulin alpha-4A chain	0.450892857
Glyceraldehyde-3-phosphate dehydrogenase	0.447761194
Tubulin alpha-1B chain	0.43902439
Profilin-1	0.435714286
14-3-3 protein zeta/delta	0.428571429
Beta-actin-like protein 2	0.412234043
Tubulin beta chain	0.412162162
Tubulin alpha-1A chain	0.405764967
Pleckstrin	0.402857143
Tubulin beta-4B chain	0.402247191
Hemoglobin subunit alpha	0.394366197
Hemoglobin subunit delta	0.387755102
Tubulin alpha-1C chain	0.38752784
Pyruvate kinase PKM	0.384180791
Transgelin-2	0.376884422
Vinculin	0.368606702
Tubulin alpha-3C chain	0.353333333
Tubulin beta-4A chain	0.342342342
Coagulation factor XIII A chain	0.340163934
Peptidyl-prolyl cis-trans isomerase A	0.339393939

Table S10. Summary of top 30 most abundant proteins in the SM-102 Liver LNP protein corona

**Protein Name**	**Abundance (NSAF)**
Actin, cytoplasmic 1	1.589333333
Actin, alpha cardiac muscle 1	0.888594164
Tubulin beta-1 chain	0.572062084
Hemoglobin subunit beta	0.557823129
Albumin	0.513957307
Profilin-1	0.471428571
Alpha-actinin-1	0.470852018
Fermitin family homolog 3	0.433283358
14-3-3 protein zeta/delta	0.432653061
Glyceraldehyde-3-phosphate dehydrogenase	0.408955224
Ras-related protein Rap-1b	0.407608696
Tubulin alpha-1B chain	0.392461197
Tubulin beta chain	0.391891892
Tubulin alpha-4A chain	0.390625
Transgelin-2	0.386934673
Cofilin-1	0.385542169
Hemoglobin subunit delta	0.37414966
Tubulin beta-4B chain	0.370786517
Tubulin alpha-1C chain	0.369710468
Hemoglobin subunit alpha	0.366197183
Tubulin alpha-1A chain	0.352549889
Pyruvate kinase PKM	0.352165725
Peptidyl-prolyl cis-trans isomerase A	0.351515152
Immunoglobulin kappa constant	0.345794393
Pleckstrin	0.342857143
Vinculin	0.333333333
Tropomyosin alpha-4 chain	0.326612903
Tubulin beta-4A chain	0.317567568
Fructose-bisphosphate aldolase A	0.31043956
Tubulin alpha-8 chain	0.307349666
